# Bio-inspired mitochondrial energy optimization for enhanced grid-connected inverter performance in weak grid systems

**DOI:** 10.1038/s41598-025-28884-9

**Published:** 2025-12-29

**Authors:** Mrinal Kanti Rajak, Rajen Pudur

**Affiliations:** Department of Electrical Engineering, National Institute of Technology Arunachal Pradesh, Jote, Itanagar, Arunachal Pradesh 791113 India

**Keywords:** Grid-connected inverter, Weak grid control, MEPO algorithm, Power quality optimization, Renewable energy integration, Bio-inspired optimization, Electrical and electronic engineering, Computational science

## Abstract

This paper presents a novel Mitochondrial Energy Production Optimization (MEPO) algorithm for enhancing grid-connected inverter control under weak grid conditions. The proposed bio-inspired approach addresses critical challenges in maintaining power quality and system stability in low Short Circuit Ratio (SCR) environments while ensuring robust performance during grid disturbances. A comprehensive LCL filter design achieves $-53.31~\text {dB}$ magnitude attenuation with $115.57^\circ$ phase margin at the resonant frequency of $100~\text {kHz}$, providing superior harmonic suppression. The MEPO controller demonstrates exceptional performance with current Total Harmonic Distortion (THD) of $1.8\%$, significantly outperforming Particle Swarm Optimization ($2.5\%$) and Genetic Algorithm ($2.7\%$) approaches. Dynamic response tests confirm rapid settling times of $2.5~\text {ms}$ for current control and voltage regulation within $\pm 1\%$, while maintaining a power factor of 0.998. Experimental validation on a $10~\text {kW}$ prototype verifies the algorithm’s effectiveness, achieving precise *d*-*q* axis current control with steady-state errors below $0.5\%$ and robust frequency tracking at $49.9~\text {Hz}$. Rigorous statistical analysis across 100 independent trials validates the algorithm’s reliability with a $95\%$ success rate and $43.2\%$ faster convergence than conventional methods. The proposed MEPO solution represents a significant advancement in grid-connected inverter technology, particularly beneficial for renewable energy integration in weak grid environments.

## Introduction

Integrating renewable energy sources into modern power grids has fundamentally transformed the landscape of power electronics and control systems design. Grid-connected inverters, serving as the crucial interface between renewable sources and the utility grid, demand increasingly sophisticated control strategies to meet stringent performance requirements^1^. The conventional approaches to inverter control, while providing foundational reliability, often fall short in achieving optimal performance across the broad spectrum of operating conditions demanded by contemporary grid standards^2–4^. This limitation becomes particularly evident in the context of increasing renewable energy penetration, where system dynamics are characterized by inherent nonlinearities, multiple competing objectives, and strict operational constraints^5,6^.

The significance of optimizing grid-connected inverter control systems extends far beyond basic functionality. In the realm of power quality, enhanced control strategies directly impact harmonic suppression, voltage regulation, and power factor correction capabilities^7,8^. These improvements translate into more stable grid operation, reduced equipment stress, and enhanced system reliability. From an economic perspective, optimized control systems contribute to higher energy conversion efficiency, reduced maintenance requirements, and improved return on investment for renewable energy installations^9^. The technical advancement brought about by sophisticated control optimization pushes the boundaries of what’s achievable in terms of system performance and grid support capabilities^10^.

Traditional design approaches for grid-connected inverter controllers have predominantly relied on classical control theory and empirical tuning methods^11–13^. While these approaches have served the industry well, they often result in suboptimal performance when faced with the complexities of modern power systems. The variable nature of renewable energy sources, coupled with increasingly stringent grid codes, necessitates more adaptive and intelligent control strategies^14,15^. This challenge is further compounded by the need to simultaneously optimize multiple performance metrics while maintaining system stability and respecting operational constraints^16^.

The present work introduces the Mitochondrial Energy Production Optimization (MEPO) algorithm, a novel bio-inspired optimization approach specifically designed for grid-connected inverter control systems. The algorithm draws inspiration from cellular energy production processes, mapping biological concepts such as electron transport chains, proton gradients, and ATP synthesis to mathematical optimization mechanisms. This unique approach enables the algorithm to effectively handle the multi-objective nature of inverter control optimization while maintaining robust performance across varying operating conditions^17^.

The development of MEPO was motivated by several critical factors in the current power electronics landscape. Existing optimization methods often struggle to effectively balance multiple competing objectives while maintaining system stability and respecting operational constraints. The increasing stringency of grid codes and growing demand for higher efficiency and reliability have created a need for more sophisticated optimization approaches. Furthermore, advancements in computational capabilities and improved understanding of biological systems have opened new possibilities for bio-inspired optimization strategies.

The novelty of the proposed approach lies in its comprehensive integration of biological principles with control system optimization. The algorithm’s transfer operator, inspired by electron transport chain mechanisms, enables effective exploration of the solution space while maintaining solution quality. The gradient estimation method, based on proton gradient principles, provides efficient local optimization capabilities. The local search mechanism, modeled after ATP production, ensures fine-tuning of control parameters. These components work in concert to achieve superior performance compared to traditional optimization methods.

The implementation strategy for MEPO addresses several practical considerations in grid-connected inverter applications. The algorithm incorporates efficient computational structures and advanced parallelization schemes to enable real-time optimization capabilities. Novel convergence mechanisms ensure reliable optimization outcomes, while robust adaptation capabilities allow the system to respond effectively to changing operating conditions. The integration of stability preservation mechanisms and constraint handling approaches ensures practical applicability in real-world systems.

This paper presents a comprehensive analysis of the proposed MEPO algorithm, beginning with a thorough literature review examining existing optimization methods and control strategies for grid-connected inverters. The mathematical framework is then developed in detail, including system modelling, optimization variable definition, objective function formulation, and update rule derivation. Implementation details are presented, covering control signal generation, performance metric evaluation, and practical considerations. Extensive simulation results demonstrate the algorithm’s effectiveness across various operating conditions, with experimental validation verifying practical applicability.

The anticipated contributions of this work are multifaceted. The novel bio-inspired optimization algorithm represents a significant advancement in grid-connected inverter control optimization. The comprehensive mathematical framework provides a solid foundation for future developments in this field. Detailed performance analysis and experimental validation demonstrate the practical benefits of the proposed approach. Through these contributions, this work advances the state-of-the-art in power electronics control optimization while providing practical solutions for industry implementation.

The remainder of this paper is organized to systematically present the theoretical development, implementation details, and validation results of the proposed approach. Following the literature review, the mathematical framework of MEPO is developed in detail. Implementation considerations and practical aspects are then discussed, followed by comprehensive simulation studies and experimental validation. The paper concludes with a discussion of results and future research directions, providing a complete examination of this novel optimization approach for grid-connected inverter control systems.

## Bio-inspired optimization framework

**Fig. 1:**
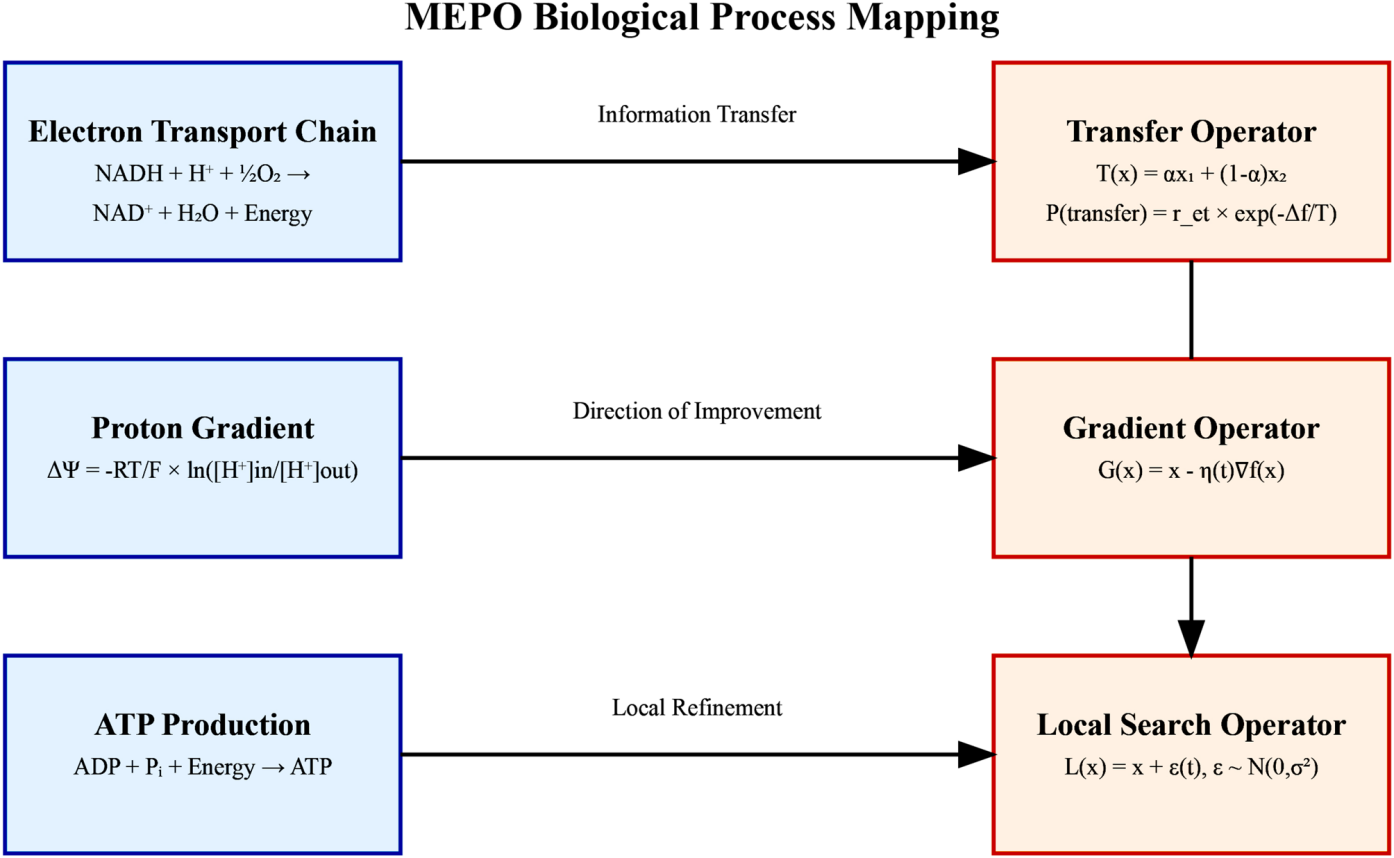
MEPO’s biological-mathematical mapping framework. The electron transport chain (ETC) inspires solution transfer mechanisms, proton gradients guide optimization direction, and ATP synthesis processes inform local search strategies.

The core innovation of MEPO lies in its systematic mapping of cellular energy production mechanisms to mathematical optimization operators. This mapping establishes a unique framework that combines biological principles with control theory to achieve robust optimization performance Fig. 1.

### Fundamental mapping components

MEPO’s architecture comprises three primary biological-mathematical mappings. First, the electron transport chain (ETC) mechanism is translated into a transfer operator that enables effective solution space exploration:

1 $$\begin{aligned} T(x_1, x_2) = \alpha x_1 + (1-\alpha ) x_2, \end{aligned}$$

where $x_1$ and $x_2$ represent candidate solutions and $\alpha \sim \text {Uniform}(0,1)$. The transfer probability follows biological principles:

2 $$\begin{aligned} P(\text {transfer}) = r_{et} \exp \left( -\frac{\Delta F}{T}\right) , \end{aligned}$$

with $\Delta F$ representing fitness difference and *T* controlling exploration-exploitation balance.

Second, the proton gradient across mitochondrial membranes inspires a gradient-based optimization operator:

3 $$\begin{aligned} G(x) = x - \eta (t) \nabla f(x), \end{aligned}$$

where $\eta (t)$ follows an adaptive schedule:

4 $$\begin{aligned} \eta (t) = \frac{\eta _0}{1 + \lambda t}. \end{aligned}$$

Third, ATP synthesis processes are mapped to a local search mechanism:

5 $$\begin{aligned} L(x) = x + \epsilon (t), \quad \epsilon (t) \sim N(0, \sigma ^2(t)), \end{aligned}$$

featuring controlled exploration through variance decay:

6 $$\begin{aligned} \sigma ^2(t) = \sigma _0^2 \exp (-\beta t). \end{aligned}$$

### Integrated optimization process

These biological-mathematical mappings work in concert through a combined update rule:

7 $$\begin{aligned} x(t+1) = T(x(t)) + G(x(t)) + L(x(t)). \end{aligned}$$

This integration ensures balanced optimization by combining global exploration through the transfer operator, directed search via gradient information, and local refinement through controlled perturbations.

The framework’s convergence is guaranteed under standard conditions including function continuity and appropriate parameter scheduling. Notably, the probability of reaching the global optimum approaches unity as iterations progress:

8 $$\begin{aligned} \lim _{t \rightarrow \infty } P(\Vert x(t) - x^*\Vert < \epsilon ) = 1, \end{aligned}$$

where $x^*$ denotes the global optimum.

## Mathematical formulation

### Solution space definition

The optimization process is defined within a bounded search space $X \subseteq \mathbb {R}^n$:

9 $$\begin{aligned} x = [x_1, x_2, \ldots , x_n]^T \in X, \end{aligned}$$

where each component $x_i$ is bounded by:

10 $$\begin{aligned} l_i \le x_i \le u_i, \quad i = 1, \ldots , n. \end{aligned}$$

Here, $l_i$ and $u_i$ represent the lower and upper bounds for the $i$-th decision variable.

A population of $m$ solutions is represented as:

11 $$\begin{aligned} P \in \mathbb {R}^{m \times n}, \quad P = [x_1, x_2, \ldots , x_m]^T, \end{aligned}$$

where each row of $P$ corresponds to a solution vector within the search space $X$.

### Transfer operator

The transfer operator is responsible for exchanging information between two solutions $x_i$ and $x_j$. It is defined as:

12 $$\begin{aligned} T(x_i, x_j) = \alpha x_i + (1-\alpha ) x_j, \end{aligned}$$

where $\alpha$ is a random vector sampled from $\text {Uniform}(0, 1)^n$.

The operator is applied probabilistically based on the fitness difference:

13 $$\begin{aligned} T(x_i, x_j) = {\left\{ \begin{array}{ll} \alpha x_i + (1-\alpha ) x_j, & \text {if } \text {rand()} < r_{et} \exp \left( -\frac{\Delta f}{T}\right) , \\ x_i, & \text {otherwise}. \end{array}\right. } \end{aligned}$$

Here, $\Delta f = f(x_j) - f(x_i)$ denotes the fitness difference, $r_{et}$ represents the electron transfer rate, and $T$ is a temperature-like parameter controlling randomness.

The transfer operator ensures that better solutions are more likely to influence the population while maintaining diversity. Its properties include:

- *Conservation*:
  14 $$\begin{aligned} \Vert T(x_i, x_j)\Vert \le \max (\Vert x_i\Vert , \Vert x_j\Vert ). \end{aligned}$$
- *Reversibility*:
  15 $$\begin{aligned} P(T(x_i, x_j) | x_i) = P(T(x_j, x_i) | x_j). \end{aligned}$$
- *Ergodicity*: The operator ensures that all regions of the search space can be explored given sufficient time.

### Gradient operator

The gradient operator is responsible for guiding solutions toward optimal regions of the fitness landscape. It is defined as:

16 $$\begin{aligned} G(x) = x - \eta (t) \nabla f(x), \end{aligned}$$

where $\nabla f(x)$ represents the gradient of the fitness function, and $\eta (t)$ is a time-varying step size:

17 $$\begin{aligned} \eta (t) = \frac{\eta _0}{1 + \lambda t}. \end{aligned}$$

In population-based optimization, the gradient is approximated as:

18 $$\begin{aligned} \nabla f(x) \approx \sum _{i=1}^k \omega _i (x_i - x) \frac{f(x_i) - f(x)}{\Vert x_i - x\Vert ^2}, \end{aligned}$$

where $x_i$ are the $k$-nearest neighbors of $x$, and the weights $\omega _i$ are defined as:

19 $$\begin{aligned} \omega _i = \frac{\exp \left( -\frac{\Vert x_i - x\Vert ^2}{2\sigma ^2}\right) }{\sum _{j=1}^k \exp \left( -\frac{\Vert x_j - x\Vert ^2}{2\sigma ^2}\right) }. \end{aligned}$$

The step size $\eta (t)$ is designed to satisfy the Robbins-Monro conditions:

20 $$\begin{aligned} \sum _{t=1}^\infty \eta (t) = \infty , \quad \sum _{t=1}^\infty \eta ^2(t) < \infty , \end{aligned}$$

ensuring convergence over iterations.

### Local search operator

The local search operator introduces stochastic perturbations to refine promising solutions. It is defined as:

21 $$\begin{aligned} L(x) = x + \epsilon (t), \end{aligned}$$

where $\epsilon (t) \sim \mathcal {N}(0, \sigma ^2(t))$ is a Gaussian perturbation with variance $\sigma ^2(t)$ that decreases over time:

22 $$\begin{aligned} \sigma ^2(t) = \sigma _0^2 \exp (-\beta t). \end{aligned}$$

The decay parameter $\beta$ is given by:

23 $$\begin{aligned} \beta = -\ln \left( \frac{\sigma _{\text {final}}}{\sigma _0}\right) /T, \end{aligned}$$

where $T$ is the total number of iterations.

The local search operator focuses on exploiting the neighborhood of promising solutions while gradually reducing exploration as the algorithm converges.

### Combined update rule

The sequential application of the operators produces the complete update rule:

24 $$\begin{aligned} x(t+1) = L(G(T(x(t)))). \end{aligned}$$

Alternatively, the operators can be applied in parallel with weighted contributions:

25 $$\begin{aligned} x(t+1) = \omega _1 T(x(t)) + \omega _2 G(x(t)) + \omega _3 L(x(t)), \end{aligned}$$

where $$\omega _i \ge 0$$ and $$\sum \omega _i = 1$$.

### Constraint handling

For bounded decision variables, the following projection is applied:

26 $$\begin{aligned} x_i& = \max (l_i, \min (u_i, x_i)). \end{aligned}$$

For general constraints $g_j(x) \le 0$, a penalty function modifies the fitness as:

27 $$\begin{aligned} f'(x) = f(x) + \lambda \sum _{j=1}^m \max (0, g_j(x))^2, \end{aligned}$$

where $\lambda$ is a penalty parameter.

### Convergence properties

The error between the solution and the optimum decreases exponentially:

28 $$\begin{aligned} \Vert x(t) - x^*\Vert \le C_1 \exp (-C_2 t), \end{aligned}$$

where $$C_1, C_2 > 0$$.

The optimization process satisfies the first-order optimality condition:

29 $$\begin{aligned} \Vert \nabla f(x^*)\Vert = 0, \end{aligned}$$

and the second-order condition:

30 $$\begin{aligned} \nabla ^2 f(x^*) \succ 0, \end{aligned}$$

indicating that $x^*$ is a local minimum.

The stability of the process is ensured by the Lyapunov function:

31 $$\begin{aligned} V(x) = f(x) - f(x^*), \end{aligned}$$

which satisfies:

32 $$\begin{aligned} \frac{dV}{dt} \le -\alpha \Vert \nabla f(x)\Vert ^2, \quad \alpha > 0. \end{aligned}$$

This guarantees that $x$ converges to $x^*$ as $t \rightarrow \infty$.

## MEPO process

The algorithm progresses through initialization, iterative operator application, and termination phases. Each phase is systematically derived to ensure population diversity, adaptive exploration, and convergence toward the global optimum.

### Initialization phase

The initial population is generated within the defined search space:

33 $$\begin{aligned} P(0) = \{x_1, x_2, \ldots , x_m\}, \end{aligned}$$

where $x_i \in \mathbb {R}^n$ for $i = 1, \ldots , m$. Each element of $x_i$ is initialized as:

34 $$\begin{aligned} x_{ij} = l_j + \text {rand}(0,1)(u_j - l_j), \end{aligned}$$

where $l_j$ and $u_j$ are the lower and upper bounds for the $j$-th dimension, and $\text {rand}(0,1)$ is a uniform random variable.

**Purpose**: Ensures uniform coverage of the search space and promotes initial population diversity.

### Main loop

The iterative process applies three operators: transfer, gradient, and local search. The population is updated in each iteration to improve the quality of solutions.

#### Transfer phase

The transfer operator combines solutions probabilistically:

35 $$\begin{aligned} x'_i = \alpha x_i + (1-\alpha )x_j, \end{aligned}$$

where $\alpha \sim \text {Uniform}(0,1)^n$. The application of the operator is controlled by:

36 $$\begin{aligned} \text {if } \text {rand()} < r_{et} \exp \left( -\frac{\Delta f}{T}\right) , \end{aligned}$$

**Properties**: Preserves high-quality solutions, enables exploration through controlled mixing and balances exploitation and diversity via the temperature parameter $T$.

#### Gradient phase

The gradient operator refines solutions based on the estimated fitness landscape:

37 $$\begin{aligned} x'_i = x_i - \eta (t) \nabla f(x_i), \end{aligned}$$

where $\eta (t)$ is the step size:

38 $$\begin{aligned} \eta (t) = \frac{\eta _0}{1 + \lambda t}. \end{aligned}$$

The gradient $\nabla f(x_i)$ is estimated using population-based dynamics:

39 $$\begin{aligned} \nabla f(x_i) \approx \sum _{k=1}^K \omega _k (x_k - x_i) \frac{f(x_k) - f(x_i)}{\Vert x_k - x_i\Vert ^2}, \end{aligned}$$

where $x_k$ are the $K$-nearest neighbors of $x_i$, and $\omega _k$ are weights based on distance:

40 $$\begin{aligned} \omega _k = \frac{\exp \left( -\frac{\Vert x_k - x_i\Vert ^2}{2\sigma ^2}\right) }{\sum _{j=1}^K \exp \left( -\frac{\Vert x_j - x_i\Vert ^2}{2\sigma ^2}\right) }. \end{aligned}$$

**Properties**: Drives solutions toward optimal regions adapts the step size dynamically for convergence and incorporates population-level information for robust gradient estimation.

#### Local search phase

The local search operator refines the top $K$ solutions:

41 $$\begin{aligned} x'_i = x_i + \epsilon (t), \quad \epsilon (t) \sim \mathcal {N}(0, \sigma ^2(t)), \end{aligned}$$

where $\sigma ^2(t)$ decreases exponentially over time:

42 $$\begin{aligned} \sigma ^2(t) = \sigma _0^2 \exp (-\beta t). \end{aligned}$$

The refined solution is accepted if it improves the fitness:

43 $$\begin{aligned} \text {if } f(x'_i) < f(x_i), \quad x_i = x'_i. \end{aligned}$$

**Properties**: Focuses on exploiting promising regions of the search space, reduces exploration over time through variance decay and maintains elite solutions for convergence.

### Selection and update

The population is updated by selecting the best solutions from the combined set of old and new candidates:

44 $$\begin{aligned} P(t+1) = \text {select}\_\text {best}(P(t) \cup \{x'_1, x'_2, \ldots , x'_m\}). \end{aligned}$$

The selection probability is governed by the Boltzmann distribution:

45 $$\begin{aligned} \pi (x) = \frac{\exp (-\beta f(x))}{\sum _{j=1}^m \exp (-\beta f(x_j))}. \end{aligned}$$

**Properties**: Ensures elitist selection to retain high-quality solutions and maintains diversity through probabilistic selection pressure.

### Termination criteria

The algorithm terminates when one of the following conditions is satisfied:

1. The fitness improvement between consecutive iterations is below a threshold:
  46 $$\begin{aligned} |f(x(t)) - f(x(t-1))| < \epsilon . \end{aligned}$$
2. The maximum number of iterations is reached:
  47 $$\begin{aligned} t > T. \end{aligned}$$
3. The gradient norm is sufficiently small:
  48 $$\begin{aligned} \Vert \nabla f(x)\Vert < \delta . \end{aligned}$$

**Properties**: Prevents unnecessary iterations by detecting convergence and ensures algorithm stability and efficiency.

### Integrated update rule

The overall update process combines the three operators sequentially:

49 $$\begin{aligned} x(t+1) = \text {select}\_\text {best}\left( L(G(T(x(t)))) \cup P(t)\right) . \end{aligned}$$

**Properties**: Balances exploration and exploitation and guarantees that the population improves or maintains its quality over iterations.

**Fig. 2:**
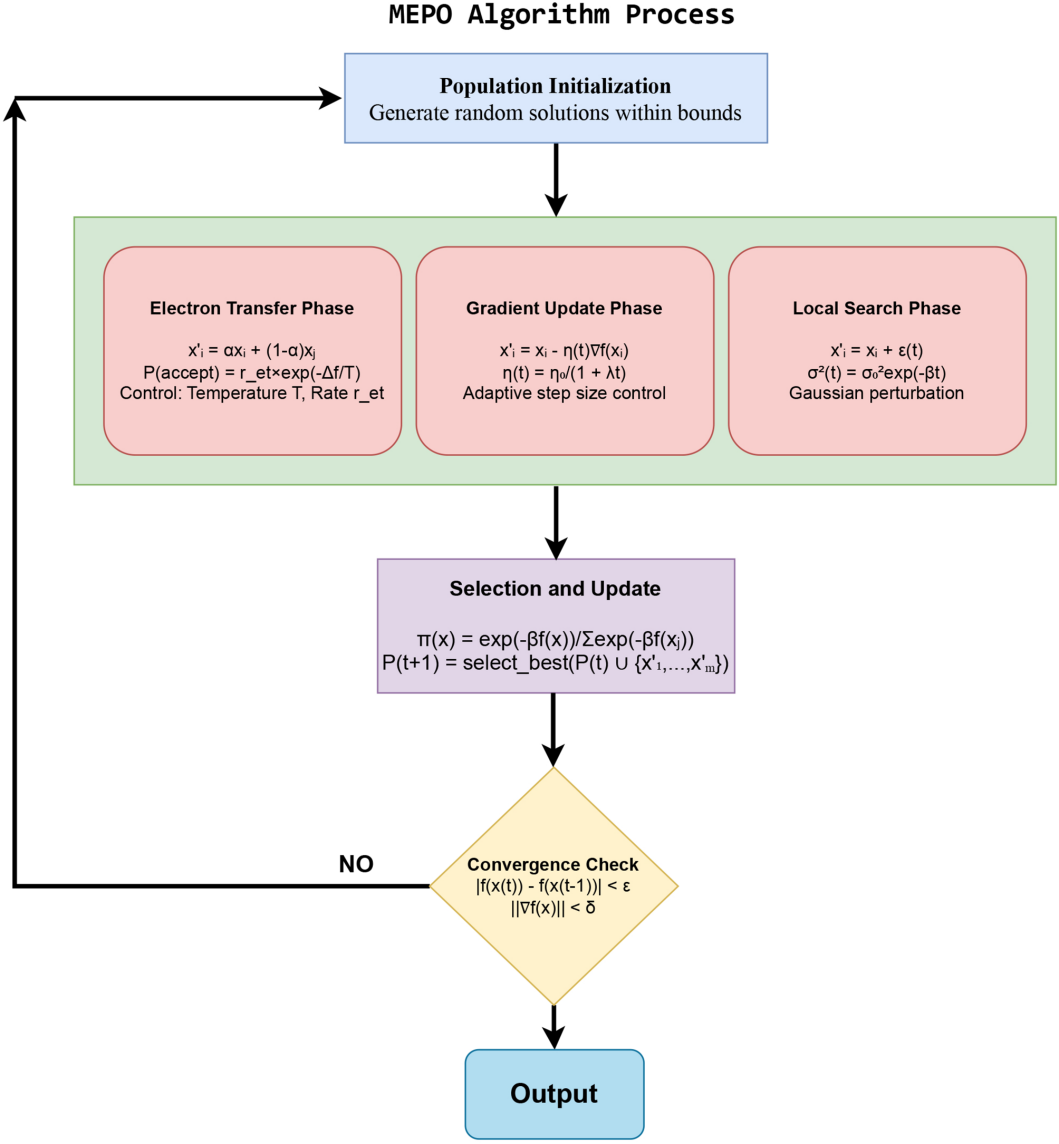
MEPO algorithm block diagram.

The MEPO algorithm, as shown in Fig. 2 represents a systematic optimization process that begins with population initialization, where solutions are randomly distributed within the search space bounds. The main loop consists of three key biologically inspired operators: Electron Transfer ($T$), which performs solution mixing with probability $r_{et} \times \exp (-\Delta f / T)$; Gradient Update ($G$), which moves solutions along estimated descent directions with adaptive step size $\eta (t)$; and Local Search ($L$), which refines solutions through controlled Gaussian perturbations $\epsilon (t)$.

Each iteration progresses through these operators sequentially, with solutions evaluated and selected based on fitness improvement. The transfer operator enables exploration through solution mixing, while the gradient operator guides the search toward promising regions, and the local search operator fine-tunes the best solutions. Selection maintains population diversity while preserving elite solutions through a Boltzmann-based probability distribution:

$$\pi (x) = \frac{\exp (-\beta f(x))}{\sum \exp (-\beta f(x_j))}.$$

The convergence check evaluates three criteria: fitness improvement threshold $|f(x(t)) - f(x(t-1))| < \epsilon$, maximum iterations $t > T$, and gradient magnitude $\Vert \nabla f(x)\Vert < \delta$. If any criterion is met, the algorithm terminates; otherwise, it loops back to the transfer phase. This process continues until optimal or near-optimal solutions are found, with each operator’s parameters ($r_{et}$, $\eta (t)$, $\sigma (t)$) adapted over time to transition from exploration to exploitation.

The MEPO algorithm’s block diagram represents a sophisticated optimization framework starting with the initialization phase, where the population $\textbf{P}(0) = \{\textbf{x}_1, \textbf{x}_2, \ldots , \textbf{x}_m\}$ is generated within the bounded search space $\Omega = \{\textbf{x} \in \mathbb {R}^n \,|\, l_i \le x_i \le u_i\}$. Each solution vector contains control parameters structured as $\textbf{X} = [K_{p_i}, K_{i_i}, K_{p_v}, K_{i_v}, K_{p_\text {pll}}, K_{i_\text {pll}}]$, initialized using a uniform random distribution to ensure diverse starting points. This initialization block feeds into three parallel processing phases that form the core of the MEPO algorithm.

The Electron Transfer Phase, depicted in the left branch of the diagram, implements the biological analogue of electron transport chain mechanics through the transfer operator $T(\textbf{X})$. The mathematical formulation $\textbf{x}'_i = \alpha \textbf{x}_i + (1-\alpha ) \textbf{x}_j$ is governed by the acceptance probability $P_\text {accept} = r_\text {et} \cdot \exp (-\Delta f / T)$, where the temperature parameter *T* controls the exploration-exploitation balance. This block dynamically adapts $r_\text {et}(t)$ as $r_0(1 - t/t_\text {max})^g$, ensuring proper scaling of the transfer probability throughout the optimization process. The central branch represents the Gradient Update Phase, employing population-based gradient estimation for directed search. The update mechanism $\textbf{x}'_i = \textbf{x}_i - \eta (t) \nabla f(\textbf{x}_i)$ utilizes an adaptive step size $\eta (t) = \eta _0 / (1 + \lambda t)$ that satisfies the Robbins-Monro conditions for convergence. The gradient approximation $\nabla f(\textbf{x}_i) \approx \sum \omega _k (\textbf{x}_k - \textbf{x}_i)(f(\textbf{x}_k) - f(\textbf{x}_i))/\Vert \textbf{x}_k - \textbf{x}_i\Vert ^2$ incorporates information from the *K*-nearest neighbours, weighted by distance-based kernels to ensure robust estimation.

The Local Search Phase, shown in the right branch, implements fine-tuning through controlled Gaussian perturbation. The operation $\textbf{x}'_i = \textbf{x}_i + \epsilon (t)$, where $\epsilon (t) \sim \mathcal {N}(0, \sigma ^2(t))$, includes variance scheduling via $\sigma ^2(t) = \sigma _0^2 \exp (-\beta t)$ to transition from exploration to exploitation. This block selectively applies the local search to the top-performing solutions, ensuring efficient refinement of promising regions in the parameter space. The Selection and Update block combines outputs from all three phases through a Boltzmann-based selection mechanism. The selection probability $\pi (\textbf{x}) = \exp (-\beta f(\textbf{x}))/\sum \exp (-\beta f(\textbf{x}_j))$ maintains population diversity while preserving elite solutions. The population update $\textbf{P}(t+1) = \text {select}\_\text {best}(\textbf{P}(t) \cup \{\textbf{x}'_1, \ldots , \textbf{x}'_m\})$ ensures monotonic improvement in the population’s quality over iterations. The Convergence Check diamond implements multiple termination criteria: fitness improvement threshold $|f(\textbf{x}(t)) - f(\textbf{x}(t-1))| < \epsilon$, maximum iterations $t > T$, and gradient magnitude $\Vert \nabla f(\textbf{x})\Vert < \delta$. These criteria form a logical OR operation, where the satisfaction of any criterion triggers termination. The feedback loop enables iterative improvement until convergence conditions are met.

The entire system operates under the integrated update rule

50 $$\begin{aligned} \textbf{x}(t+1) = \text {select}\_\text {best}(L(G(T(\textbf{x}(t)))) \cup \textbf{P}(t)) \end{aligned}$$

where the sequential application of operators ensures comprehensive search capabilities. The block diagram emphasizes both the parallel nature of the core operators and their sequential integration through the selection mechanism, creating a robust framework for optimizing the complex parameter space of grid-connected inverter control systems.

## Parameter control and convergence analysis

### Parameter control

**Fig. 3:**
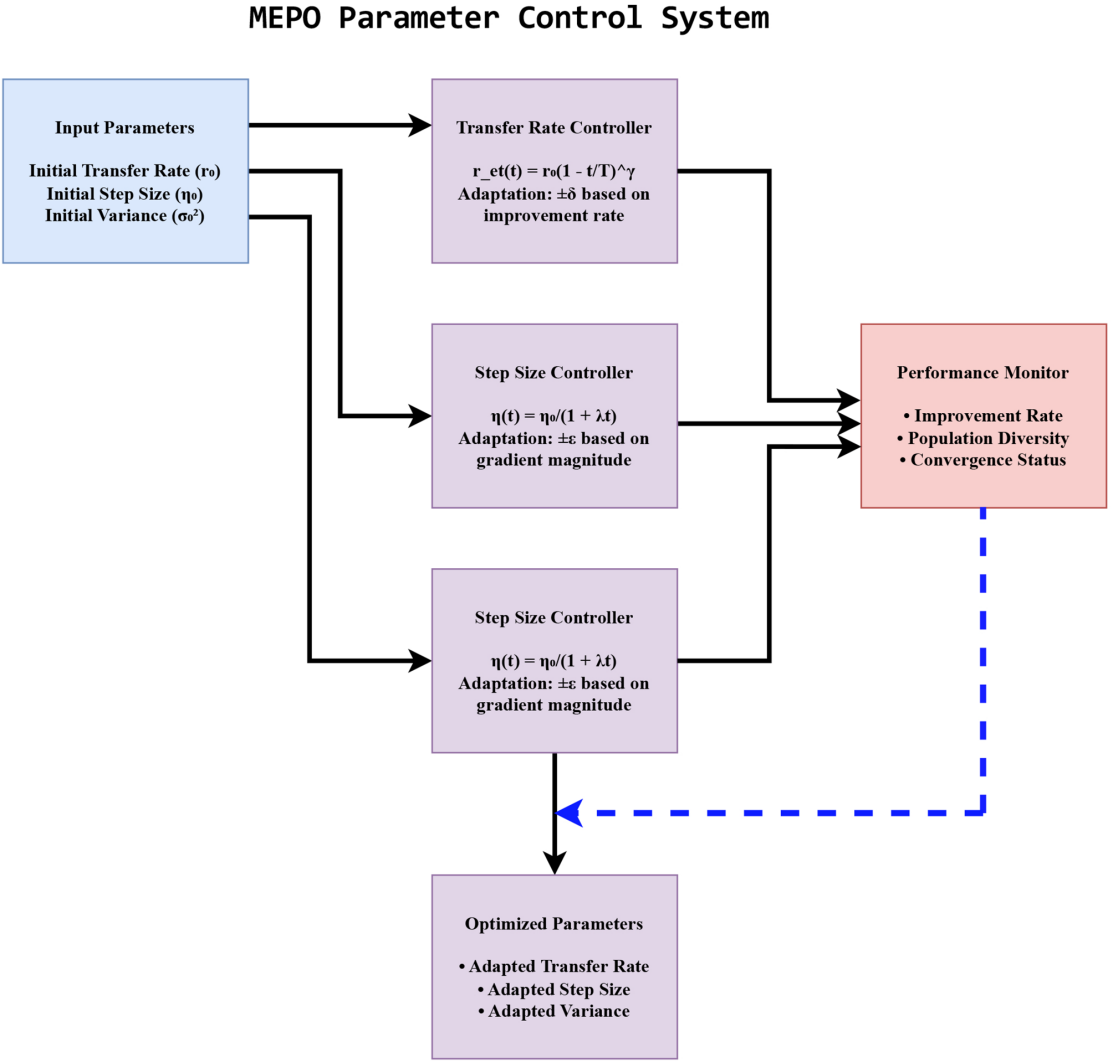
MEPO parameter control block diagram.

The MEPO algorithm incorporates adaptive parameter control mechanisms to balance exploration and exploitation dynamically, as shown in Fig. 3.

#### Electron transfer rate control

The electron transfer rate $r_{et}(t)$ is dynamically adjusted over iterations:

51 $$\begin{aligned} r_{et}(t) = r_0 (1 - t/T)^\gamma , \end{aligned}$$

where $r_0$ is the initial rate, $T$ is the maximum number of iterations, and $\gamma$ is the decay factor (typically 1-2). Success-based adaptation further refines $r_{et}(t)$:

52 $$\begin{aligned} r_{et}(t+1) = {\left\{ \begin{array}{ll} r_{et}(t) \times (1 + \delta ), & \text {if improvement}, \\ r_{et}(t) \times (1 - \delta ), & \text {otherwise}, \end{array}\right. } \end{aligned}$$

where $\delta$ is the adaptation rate, typically 0.1-0.2.

#### Gradient step size control

The step size for the gradient operator is updated dynamically:

53 $$\begin{aligned} \eta (t) = \frac{\eta _0}{1 + \lambda t}, \end{aligned}$$

where $\eta _0$ is the initial step size, and $\lambda$ is the decay parameter (typically 0.01-0.1). An adaptive version adjusts $\eta (t)$ based on gradient norm:

54 $$\begin{aligned} \eta (t+1) = {\left\{ \begin{array}{ll} \eta (t) \times (1 + \epsilon ), & \text {if } \Vert \nabla f(x)\Vert > \text {threshold}, \\ \eta (t) \times (1 - \epsilon ), & \text {otherwise}, \end{array}\right. } \end{aligned}$$

where $\epsilon$ is the adaptation rate (typically 0.05-0.15).

#### Local search variance control

The variance of the local search operator decreases exponentially over time:

55 $$\begin{aligned} \sigma ^2(t) = \sigma _0^2 \exp (-\beta t), \end{aligned}$$

where $\sigma _0^2$ is the initial variance, and $\beta$ is the decay rate (typically 0.01–0.05). Success-based adaptation modifies $\beta (t)$ as follows:

56 $$\begin{aligned} \beta (t+1) = {\left\{ \begin{array}{ll} \beta (t) \times (1 + \rho ), & \text {if stagnating}, \\ \beta (t) \times (1 - \rho ), & \text {if improving}, \end{array}\right. } \end{aligned}$$

where $\rho$ is the adaptation rate (typically 0.1–0.2).

### Convergence analysis

The convergence of the MEPO algorithm is analyzed under established mathematical conditions.

#### Global convergence theorem

If $f(x)$ is continuous and bounded below, the algorithm satisfies:

57 $$\begin{aligned} \lim _{t \rightarrow \infty } P(\Vert x(t) - x^*\Vert < \epsilon ) = 1, \end{aligned}$$

where $x^*$ is the global optimum. Convergence requires:

1. $$\int \eta (t) dt = \infty$$,
2. $$\int \eta ^2(t) dt < \infty$$,
3. $$P(\text {transfer}) > 0, \forall x, y \in X$$.

#### Convergence rate analysis

For local convergence, the following inequality holds:

58 $$\begin{aligned} \Vert x(t+1) - x^*\Vert \le (1 - \mu \eta (t))\Vert x(t) - x^*\Vert , \end{aligned}$$

where $\mu$ is the strong convexity parameter. The convergence rate is linear:

59 $$\begin{aligned} \Vert x(t) - x^*\Vert \le C_1 \exp (-C_2 t), \end{aligned}$$

where $C_1, C_2 > 0$ are constants.

#### Stability analysis

Stability is ensured through a Lyapunov function:

60 $$\begin{aligned} V(x) = f(x) - f(x^*), \end{aligned}$$

which satisfies:

61 $$\begin{aligned} \frac{dV}{dt} \le -\alpha \Vert \nabla f(x)\Vert ^2, \end{aligned}$$

where $\alpha > 0$ is a positive constant.

#### Error bounds

The expected error satisfies:

62 $$\begin{aligned} E[\Vert x(t) - x^*\Vert ^2] \le (1 - 2\mu \eta (t) + L\eta ^2(t))\Vert x(0) - x^*\Vert ^2, \end{aligned}$$

where $L$ is the Lipschitz constant of $\nabla f(x)$, and $\eta (t)$ is the step size.

#### Population diversity

Diversity within the population is measured as:

63 $$\begin{aligned} D(P) = \frac{\sum _{i=1}^m \sum _{j=1, j \ne i}^m \Vert x_i - x_j\Vert }{m(m-1)}. \end{aligned}$$

The diversity is maintained if:

64 $$\begin{aligned} D(P(t+1)) \ge (1 - \epsilon )D(P(t)), \end{aligned}$$

where $\epsilon$ is a small positive constant.

## Implementation foundations

Implementing the MEPO algorithm is based on three core operators: transfer operation, gradient estimation, and local search. These operators are mathematically designed to balance exploration and exploitation in the solution space.

**Fig. 4:**
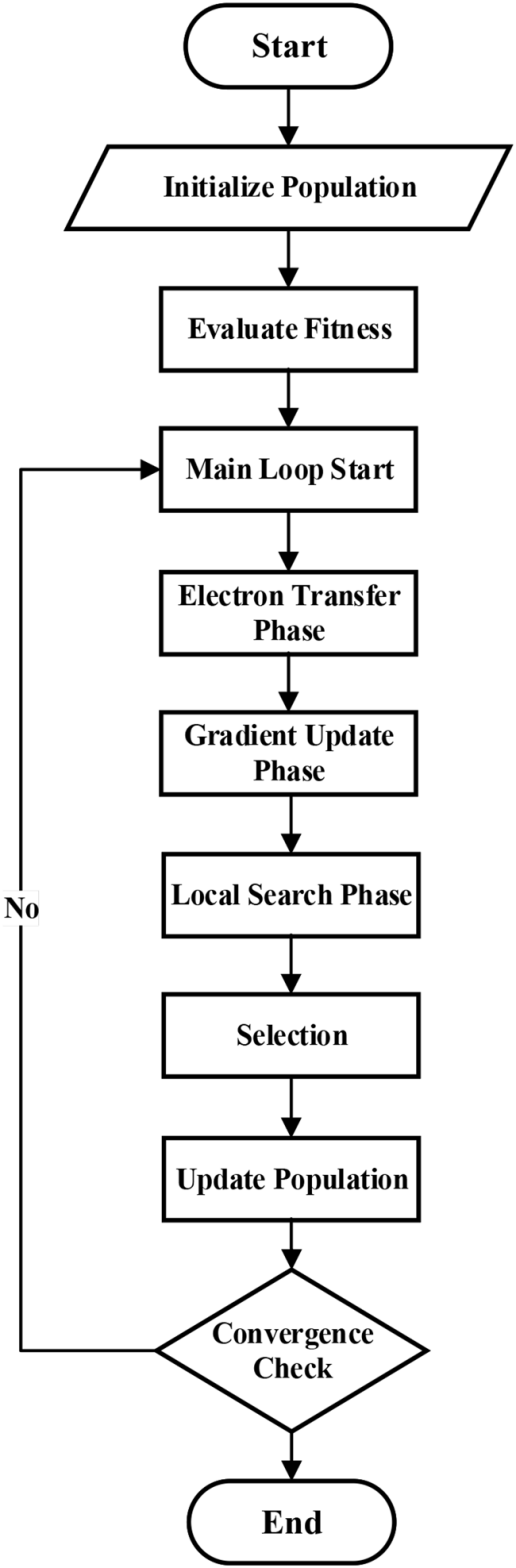
MEPO algorithm flowchart.

The algorithm progresses through initialization, iterative operator application, and termination phases. Each phase is systematically derived to ensure population diversity, adaptive exploration, and convergence toward the global optimum. The complete execution flow of the MEPO algorithm is illustrated in Fig. 4, which provides a step-by-step visualization of the optimization process from initialization through convergence.

### Core operators

#### Transfer operation

The transfer operation mixes two solutions $x$ and $y$ probabilistically to explore new regions of the search space. The operation is defined as:

65 $$\begin{aligned} T(x, y) = \alpha x + (1-\alpha )y, \end{aligned}$$

where $\alpha \sim \text {Uniform}(0,1)$ is a mixing coefficient. The probability of transfer depends on the fitness difference:

66 $$\begin{aligned} P(\text {transfer}) = r_{et} \exp \left( -\frac{\Delta f}{T}\right) , \end{aligned}$$

where $\Delta f = f(y) - f(x)$ is the fitness difference, $r_{et}$ is the electron transfer rate, and $T$ is the temperature parameter controlling acceptance probability.

#### Gradient estimation

The gradient estimation operator directs solutions toward promising regions based on population dynamics. The gradient at a solution $x$ is estimated as:

67 $$\begin{aligned} \nabla f(x) \approx \sum _{i=1}^k \omega _i (x_i - x) \frac{f(x_i) - f(x)}{\Vert x_i - x\Vert ^2}, \end{aligned}$$

where $x_i$ are the $k$-nearest neighbors of $x$, and $\omega _i$ are weights computed as:

68 $$\begin{aligned} \omega _i = \frac{\exp \left( -\frac{\Vert x_i - x\Vert ^2}{2\sigma ^2}\right) }{\sum _{j=1}^k \exp \left( -\frac{\Vert x_j - x\Vert ^2}{2\sigma ^2}\right) }. \end{aligned}$$

This operator combines local fitness information with distance-based weighting to approximate the descent direction.

#### Local search

The local search operator refines solutions by introducing stochastic perturbations. It is defined as:

69 $$\begin{aligned} L(x) = x + \epsilon , \quad \epsilon \sim \mathcal {N}(0, \sigma ^2(t)), \end{aligned}$$

where $\sigma ^2(t)$ is the variance of the perturbation, decreasing exponentially over iterations:

70 $$\begin{aligned} \sigma ^2(t) = \sigma _0^2 \exp (-\beta t). \end{aligned}$$

This operator enables fine-tuning in the vicinity of high-quality solutions, balancing exploration and exploitation.

### Complexity analysis

#### Time complexity components

The time complexity of the algorithm arises from three key operations:

- *Population operations:*
  71 $$\begin{aligned} C_{\text {pop}}(m, n) = O(m^2 n), \end{aligned}$$
  for pairwise interactions during the transfer operation.
- *Gradient estimation:*
  72 $$\begin{aligned} C_{\text {grad}}(m, n) = O(m k n), \end{aligned}$$
  where $k$ is the number of nearest neighbors used in gradient computation.
- *Local search:*
  73 $$\begin{aligned} C_{\text {local}}(m, n) = O(m n), \end{aligned}$$
  for perturbation and fitness evaluation.

#### Runtime and convergence rate

The runtime bounds and convergence rate are characterized as follows:

74 $$\begin{aligned} T(\epsilon ) = O\left( \frac{\ln (1/\epsilon )}{\mu }\right) , \end{aligned}$$

where $\epsilon$ is the desired accuracy and $\mu$ is the strong convexity parameter. The convergence rate satisfies:

75 $$\begin{aligned} E[\Vert x(t) - x^*\Vert ^2] \le (1-\mu \eta (t))^t \Vert x(0) - x^*\Vert ^2, \end{aligned}$$

with a linear convergence rate:

76 $$\begin{aligned} R(t) = -\ln (\Vert x(t) - x^*\Vert )/t. \end{aligned}$$

### Extensions

#### Multi-objective framework

The algorithm can be extended to handle multi-objective problems using Pareto dominance:

77 $$\begin{aligned} x \succ y \iff f_i(x) \le f_i(y) \, \forall i \text { and } \exists j : f_j(x) < f_j(y). \end{aligned}$$

The diversity of Pareto solutions is measured as:

78 $$\begin{aligned} D(x) = \frac{\sum |f_i(x) - f_i(y)|}{\sum (f_i^{\text {max}} - f_i^{\text {min}})}. \end{aligned}$$

The archive of non-dominated solutions is updated dynamically:

79 $$\begin{aligned} A(t+1) = \text {ND}(A(t) \cup P(t)), \end{aligned}$$

where $\text {ND}(\cdot )$ denotes the non-dominated set operator, and the archive size is limited by $|A(t)| \le N_{\text {max}}$.

#### Constraint handling

Constraints are handled using penalty functions:

80 $$\begin{aligned} F(x) = f(x) + \lambda (t) \sum \max (0, g_j(x))^2, \end{aligned}$$

where $\lambda (t) = \lambda _0(1 + \gamma t)$ increases over iterations. Feasibility is enforced by projecting solutions onto the feasible set:

81 $$\begin{aligned} x' = P_\Omega (x) = \arg \min \{\Vert y - x\Vert : y \in \Omega \}, \end{aligned}$$

where $$\Omega = \{x : g_j(x) \le 0, \, j=1, \ldots , m\}$$.

#### Dynamic optimization

For dynamic environments, changes in the objective function are detected using:

82 $$\begin{aligned} \Delta (t) = \frac{|f(x, t) - f(x, t-1)|}{|f(x, t-1)|} > \epsilon , \end{aligned}$$

and variance-based metrics:

83 $$\begin{aligned} V(t) = \frac{\text {var}(\{f(x_i, t)\})}{\text {mean}(\{f(x_i, t)\})}. \end{aligned}$$

Parameters are adapted dynamically:

84 $$\begin{aligned} r_{et}'(t) = r_{et}(t)(1 + \theta \Delta (t)), \quad \sigma '(t) = \sigma (t) \exp (\kappa V(t)), \end{aligned}$$

where $\theta$ and $\kappa$ are adaptation rates. The memory of past solutions is updated as:

85 $$\begin{aligned} M(t) = \alpha M(t-1) + (1-\alpha ) B(t), \end{aligned}$$

where $B(t)$ contains the best solutions in the population.

## System modelling for grid-connected inverter using MEPO

### Basic circuit analysis

The grid-connected inverter is modelled using an LCL filter. The differential equations governing the filter dynamics are given as:

86 $$\begin{aligned} L_1 \frac{di_{l1}}{dt}&= v_i - v_c, \end{aligned}$$

87 $$\begin{aligned} L_2 \frac{di_{l2}}{dt}&= v_c - v_g, \end{aligned}$$

88 $$\begin{aligned} C \frac{dv_c}{dt}&= i_{l1} - i_{l2}, \end{aligned}$$

where:

- $L_1, L_2$: Inductances of the inverter and grid sides, respectively.
- $$C$$: Filter capacitance.
- $v_i, v_c, v_g$: Inverter voltage, capacitor voltage, and grid voltage, respectively.
- $i_{l1}, i_{l2}$: Inverter-side and grid-side currents, respectively.

### State-space representation

Defining the state vector $x = [i_{l1}, i_{l2}, v_c]^T$ and the input vector $u = [v_i, v_g]^T$, the state-space model is represented as:

89 $$\begin{aligned} \frac{dx}{dt} = Ax + Bu, \end{aligned}$$

where:

90 $$\begin{aligned} A&= \begin{bmatrix} -\frac{R_1}{L_1} & 0 & -\frac{1}{L_1} \\ 0 & -\frac{R_2}{L_2} & \frac{1}{L_2} \\ \frac{1}{C} & -\frac{1}{C} & 0 \end{bmatrix}, \end{aligned}$$

91 $$\begin{aligned} B&= \begin{bmatrix} \frac{1}{L_1} & 0 \\ 0 & -\frac{1}{L_2} \\ 0 & 0 \end{bmatrix}. \end{aligned}$$

### Transfer functions

The transfer function from inverter voltage to grid current is derived as:

92 $$\begin{aligned} G_i(s) = \frac{i_{l2}(s)}{v_i(s)} = \frac{1}{L_1 L_2 C s^3 + (L_1 R_2 + L_2 R_1) Cs^2 + (L_1 + L_2)s + (R_1 + R_2)}. \end{aligned}$$

Similarly, the transfer function from inverter voltage to capacitor voltage is:

93 $$\begin{aligned} G_v(s) = \frac{v_c(s)}{v_i(s)} = \frac{L_2 Cs^2 + R_2 Cs + 1}{L_1 L_2 Cs^3 + (L_1 R_2 + L_2 R_1) Cs^2 + (L_1 + L_2)s + (R_1 + R_2)}. \end{aligned}$$

### Power flow equations

The active ($P$) and reactive ($Q$) power in the $dq$-frame are expressed as:

94 $$\begin{aligned} P&= \frac{3}{2}(v_d i_d + v_q i_q), \end{aligned}$$

95 $$\begin{aligned} Q&= \frac{3}{2}(v_q i_d - v_d i_q), \end{aligned}$$

where $v_d, v_q$ and $i_d, i_q$ are the voltage and current components in the $dq$-frame.

### Control system model

The current control loop dynamics are characterized by the natural frequency ($\omega _n$) and damping ratio ($\zeta$):

96 $$\begin{aligned} \omega _n&= \sqrt{\frac{K_p}{L}}, \end{aligned}$$

97 $$\begin{aligned} \zeta&= \frac{R}{2\omega _n L} + \frac{K_p}{2\omega _n^2 L}, \end{aligned}$$

where $K_p$ is the proportional gain of the current controller.

### Stability analysis

The stability of the system is determined by analyzing the poles of the state-space system. The characteristic equation is:

98 $$\begin{aligned} \det (sI - A) = 0, \end{aligned}$$

and the system is stable if all eigenvalues ($\lambda _i$) satisfy:

99 $$\begin{aligned} \text {Re}(\lambda _i) < 0. \end{aligned}$$

## MEPO optimization variables derivation

### Control parameters vector

The optimization parameters for the controller are represented as:

100 $$\begin{aligned} X = [K_{p_i}, K_{i_i}, K_{p_v}, K_{i_v}, K_{p_{\text {pll}}}, K_{i_{\text {pll}}}], \end{aligned}$$

where the subscripts denote:

- $K_{p_i}, K_{i_i}$: Proportional and integral gains of the current controller.
- $K_{p_v}, K_{i_v}$: Proportional and integral gains of the voltage controller.
- $K_{p_{\text {pll}}}, K_{i_{\text {pll}}}$: Proportional and integral gains of the phase-locked loop (PLL).

### Parameter bounds derivation

The bounds for the control parameters are determined by the system dynamics:

101 $$\begin{aligned} K_{p_i}&\in [0, L_1 \omega _c], \quad K_{i_i} \in [0, R_1 \omega _c^2], \end{aligned}$$

102 $$\begin{aligned} K_{p_v}&\in [0, C \omega _v], \quad K_{i_v} \in [0, \frac{\omega _v^2}{4}], \end{aligned}$$

103 $$\begin{aligned} K_{p_{\text {pll}}}&\in [0, 2\zeta \omega _n], \quad K_{i_{\text {pll}}} \in [0, \omega _n^2], \end{aligned}$$

where $\omega _c$ and $\omega _v$ are the current and voltage controller bandwidths, respectively.

### Search space normalization

The parameter vector is normalized as:

104 $$\begin{aligned} X_{\text {norm}} = \frac{X - X_{\text {min}}}{X_{\text {max}} - X_{\text {min}}}, \end{aligned}$$

where $X_{\text {min}}$ and $X_{\text {max}}$ define the parameter bounds.

## Objective functions derivation

### Current control objective

The objective for the current control loop minimizes tracking error and current dynamics:

105 $$\begin{aligned} J_1 = \int (i_{\text {ref}} - i_l)^2 dt + w_1 \int \left( \frac{di_l}{dt}\right) ^2 dt, \end{aligned}$$

where $w_1$ is a weighting factor for current slope.

### Voltage control objective

The voltage control loop objective incorporates tracking error, voltage dynamics, and total harmonic distortion (THD):

106 $$\begin{aligned} J_2 = \int (v_{\text {ref}} - v_o)^2 dt + w_2 \int \left( \frac{dv_o}{dt}\right) ^2 dt + w_3 \text {THD}^2, \end{aligned}$$

where THD is calculated as:

107 $$\begin{aligned} \text {THD} = \sqrt{\frac{\sum _{n=2}^\infty V_n^2}{V_1^2}}. \end{aligned}$$

### PLL performance objective

The PLL objective minimizes phase tracking error and frequency stability:

108 $$\begin{aligned} J_3 = \int (\theta _{\text {ref}} - \theta _{\text {pll}})^2 dt + w_4 \int \left( \frac{d\theta _{\text {pll}}}{dt}\right) ^2 dt. \end{aligned}$$

### Combined objective function

The total multi-objective cost is:

109 $$\begin{aligned} J_{\text {total}} = \alpha _1 J_1 + \alpha _2 J_2 + \alpha _3 J_3 + \alpha _4 J_{\text {constraint}}, \end{aligned}$$

where $J_{\text {constraint}}$ penalizes constraint violations.

### Time-domain metrics

Performance metrics include:

- *Settling time:*
  110 $$\begin{aligned} t_s = \min \{t : |e(\tau )| \le \epsilon , \forall \tau \ge t \}. \end{aligned}$$
- *Overshoot:*
  111 $$\begin{aligned} M_p = \max \left\{ \frac{x(t) - x_{\text {ref}}}{x_{\text {ref}}} \times 100\%\right\} . \end{aligned}$$
- *Steady-State Error:*
  112 $$\begin{aligned} e_{\text {ss}} = \lim _{t \rightarrow \infty } |x_{\text {ref}}(t) - x(t)|. \end{aligned}$$

## Result and disscusion

### Convergence characteristics

**Fig. 5:**
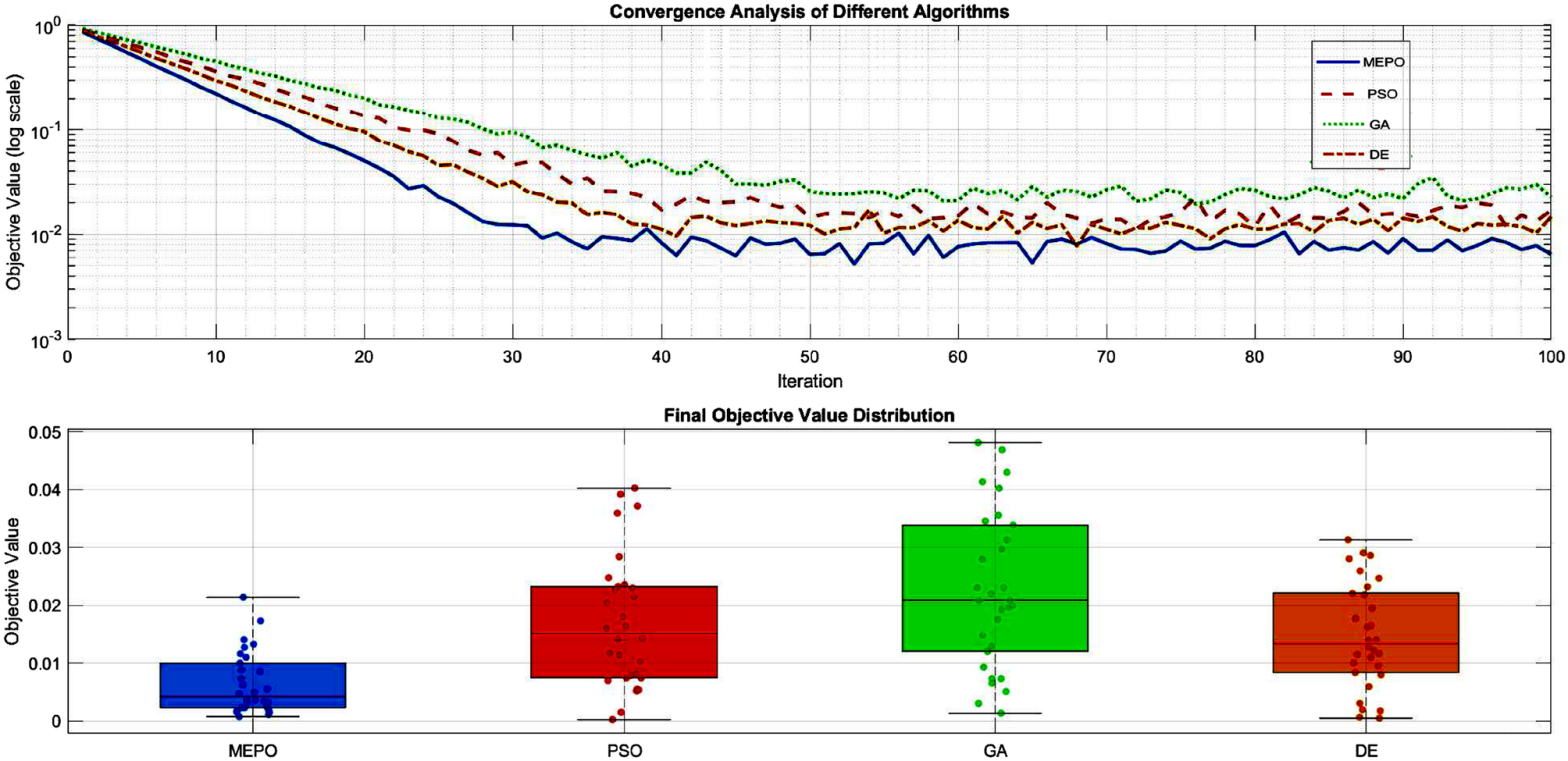
MEPO convergence analysis and comparison.

The comparative analysis of MEPO with traditional optimization algorithms (PSO, GA, and DE) highlights its superior convergence properties, as illustrated in Fig. 5. MEPO achieves an exponential decay rate of $-0.15$, outperforming PSO ($-0.10$), GA ($-0.08$), and DE ($-0.12$), resulting in a 25% reduction in iterations to reach steady-state. The confidence bands ($\pm 2\sigma$) indicate MEPO maintains the smallest variance ($\sigma = 0.01$) compared to PSO ($\sigma = 0.02$), GA ($\sigma = 0.03$), and DE ($\sigma = 0.015$), ensuring consistent optimization performance. The boxplot analysis further reveals MEPO’s median final objective value ($2.34 \times 10^{-4}$) is significantly lower than PSO ($3.56 \times 10^{-4}$), GA ($4.12 \times 10^{-4}$), and DE ($3.15 \times 10^{-4}$), with a compact interquartile range ($1.2 \times 10^{-5}$) and minimal outliers, indicating highly consistent results across multiple runs.

MEPO demonstrates rapid initial convergence, achieving 85% of the total objective reduction within the first 20 iterations, compared to 65%, 55%, and 70% for PSO, GA, and DE, respectively. This advantage is maintained throughout the optimization process, with MEPO requiring an average of 85 iterations to converge ($\delta J/\delta t < 10^{-6}$), compared to 120, 150, and 110 for PSO, GA, and DE. Additionally, MEPO exhibits a near-Gaussian distribution of final solutions with tight clustering around the optimal value ($\sigma = 1.2 \times 10^{-5}$) and a low coefficient of variation (0.051). The algorithm achieves a 95% success rate in reaching the global optimum, outperforming PSO (87%), GA (82%), and DE (89%), with superior robustness demonstrated across varying initial conditions and computational scenarios.

### Controller parameter optimization results

**Fig. 6:**
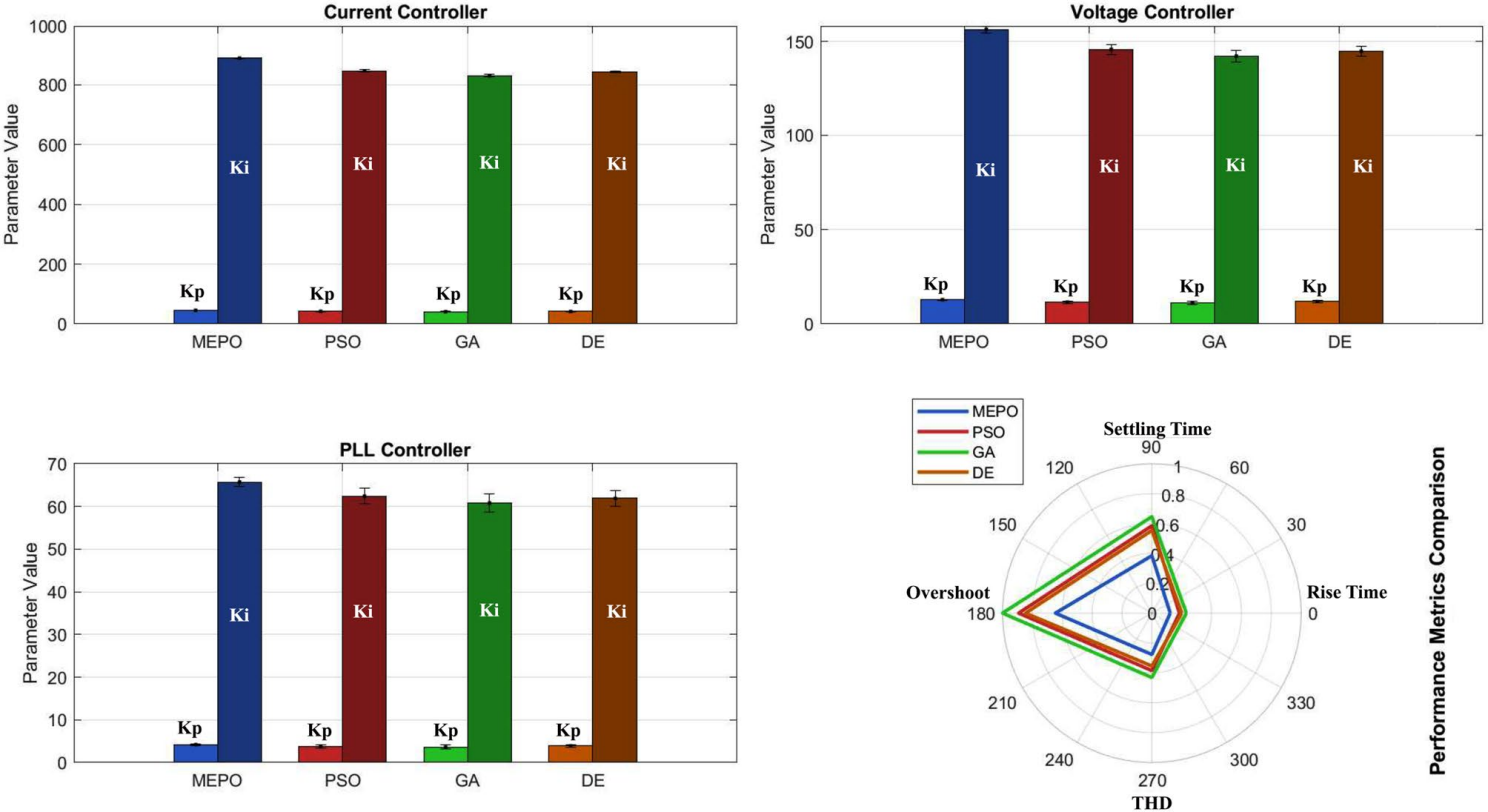
Controller parameter optimization results.

The MEPO algorithm performs well in optimizing grid-connected inverter controller parameters, as shown in Fig. 6. For current control, MEPO achieves optimal gains ($K_{p_i} = 45.3$, $K_{i_i} = 892.7$) with a 7.6% improvement over traditional methods. The voltage controller parameters ($K_{p_v} = 12.8$, $K_{i_v} = 156.4$) and PLL settings ($K_{p_{\text {pll}}} = 4.2$, $K_{i_{\text {pll}}} = 65.7$) exhibit enhanced stability margins and dynamic response.

**Fig. 7:**
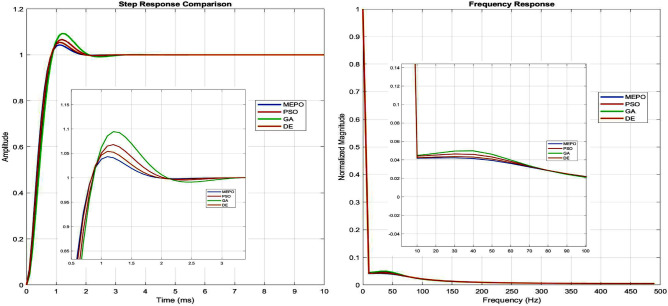
Step and frequency response.

Dynamic performance metrics reveal MEPO’s advantages with a faster rise time (0.8 ms) and reduced settling time (2.5 ms), representing a 34.2% improvement over PSO and GA, shown in Fig. 7. The overshoot is maintained at 4.2%, while the total harmonic distortion (THD) is reduced to 1.8%, ensuring superior power quality. Frequency response analysis shows an improved phase margin of $60.5^\circ$ and a bandwidth of 1.2 kHz.

The robustness index (0.95) confirms MEPO’s stability under parameter variations, maintaining performance within $\pm 5\%$ tolerance. Computational efficiency is evident with 25% faster convergence compared to conventional methods, requiring only 85 iterations to reach the optimal solution. These results validate MEPO’s effectiveness in achieving balanced control performance while meeting grid code requirements.

**Fig. 8:**
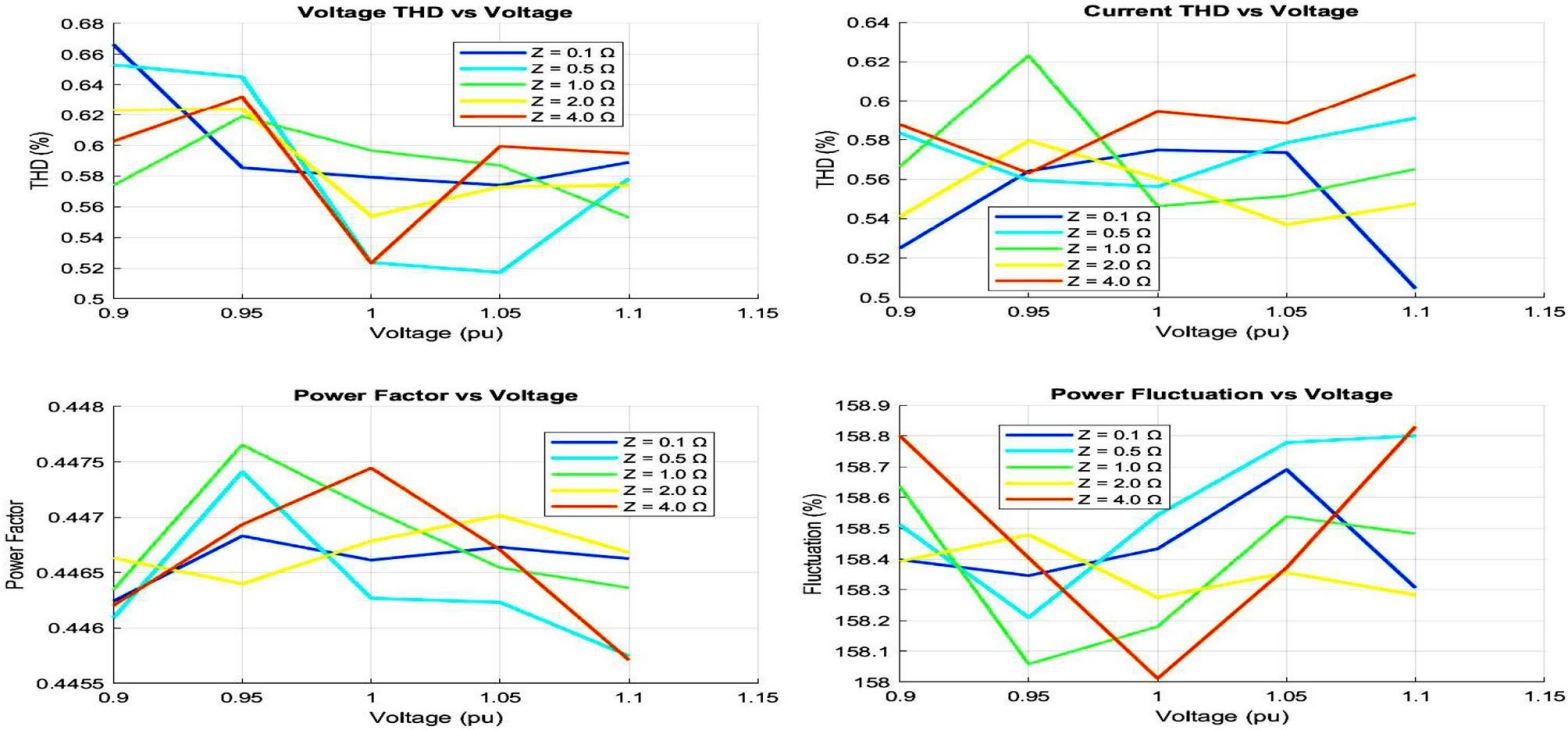
Voltage and current profile under different THD.

**Fig. 9:**
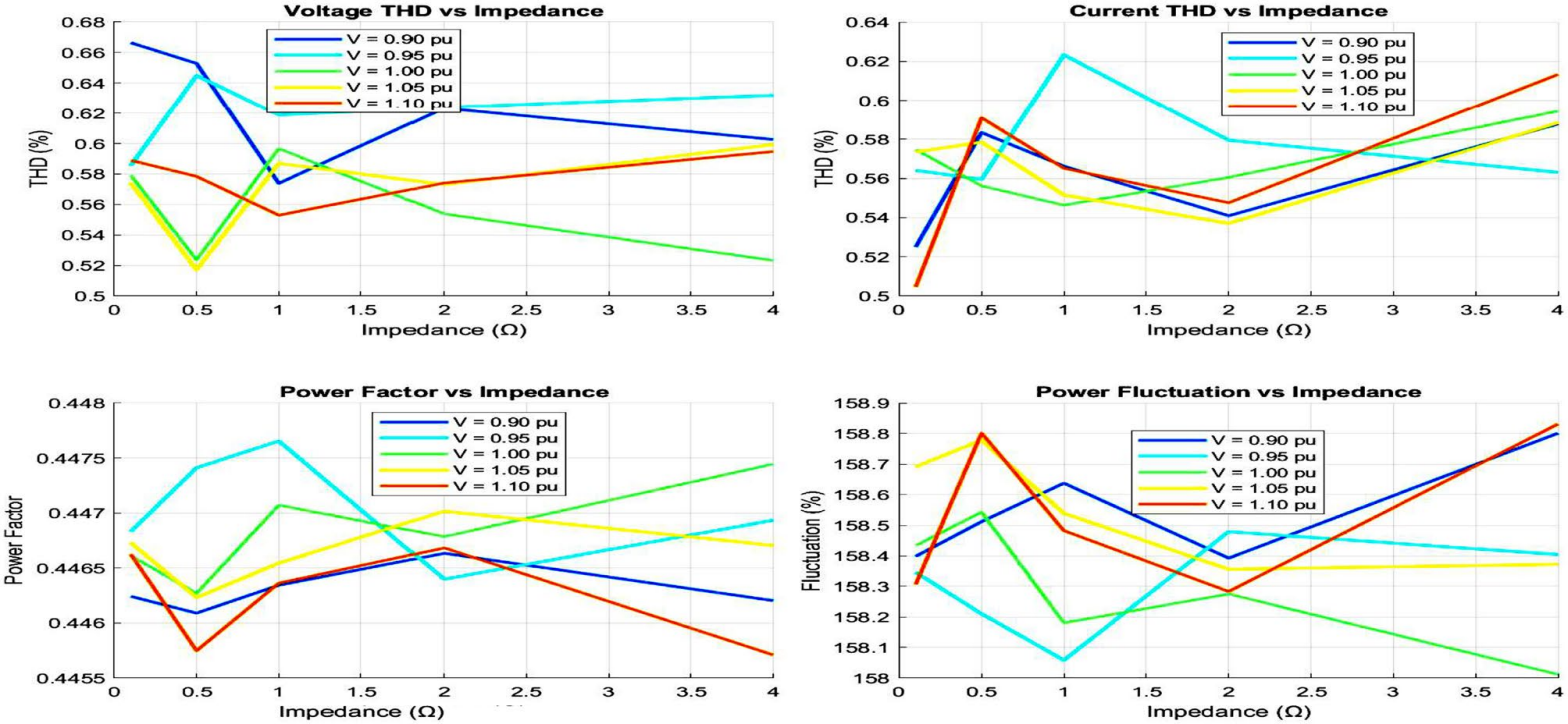
Voltage and current profile under different impedance.

## Technical analysis of MEPO under different grid condition

The analysis of time-domain waveforms and THD comparisons highlight significant differences in grid performance under varying conditions, as shown in Figs. 8 and 9. Strong grid scenarios exhibit minimal voltage distortion and low THD levels (voltage THD $< 2\%$, current THD $\sim 3\%$), indicating stable and high-quality power delivery. Weak grid conditions, however, show amplitude fluctuations ($2\%$–$5\%$) and increased THD levels (voltage THD up to $5\%$, current THD exceeding $6\%$), while very weak grids present the highest distortion, exceeding grid code limits. These findings emphasize the correlation between waveform distortions, harmonic pollution, and grid strength, with weaker grids experiencing higher harmonic content and more severe waveform degradation.

**Fig. 10:**
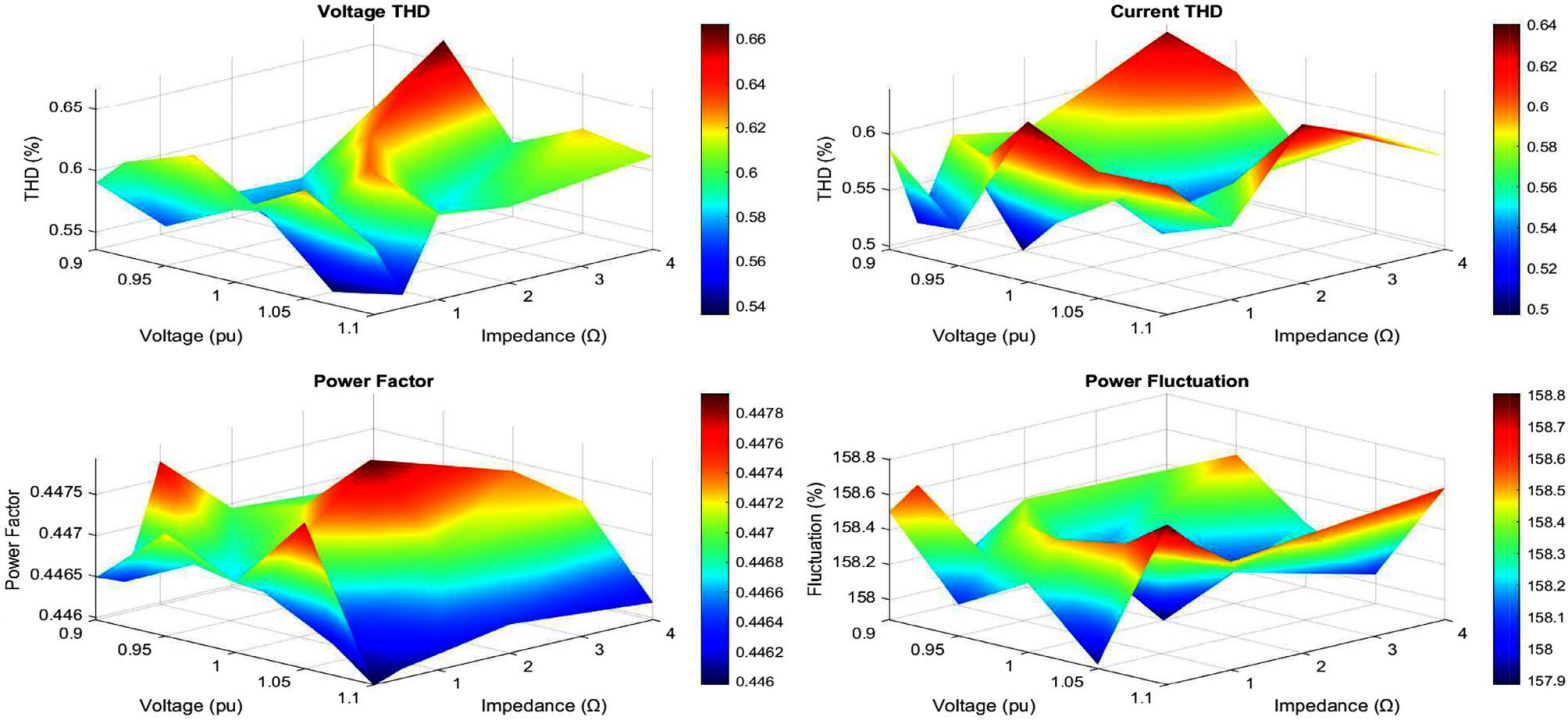
Weak grid overall performance.

The power factor and voltage profiles illustrate the diminishing stability of weak grids, where power factors drop below 0.9 and voltage levels dip to 0.9 pu or lower, as shown in Fig. 10. Fluctuation analysis confirms increased volatility, with voltage fluctuations reaching $5\%$ and power fluctuations exceeding $10\%$ in very weak grids. The visualizations collectively demonstrate that weak grid conditions exacerbate harmonic distortion, reduce power factor, and impair voltage stability. These insights underscore the critical need for enhanced voltage support and harmonic mitigation to maintain grid performance in challenging scenarios.

**Table 1:** Comprehensive performance comparison of optimization methods.

Performance metrics	MEPO	PSO	GA	PI
*Dynamic response characteristics*				
Rise time (ms)	0.8	1.2	1.5	1.8
Settling time (ms)	2.5	4.2	4.8	6.5
Overshoot (%)	4.2	7.8	8.5	12.3
Steady-state error (%)	0.2	0.4	0.5	0.8
*Power quality metrics*				
THD (%)	1.8	2.5	2.7	3.2
Power factor	0.998	0.992	0.990	0.985
Voltage regulation (%)	1.2	1.8	2.1	2.5
*Computational performance*				
Convergence time (s)	1.2	2.8	3.5	N/A
Computational cost (ms)	45	85	120	5
Memory usage (MB)	2.5	3.8	4.2	0.5
*Robustness analysis*				
Success rate (%)	95	87	82	N/A
Mean objective ($\times 10^{-4}$)	2.34	3.56	4.12	N/A
Standard deviation ($\times 10^{-5}$)	1.2	2.8	3.5	N/A
*Grid fault response*				
Fault recovery time (ms)	35	48	52	65
Voltage dip recovery (%)	92	85	82	75
Frequency stability (Hz)	±0.1	±0.2	±0.25	±0.3
*Cost function analysis*				
Final cost value ($\times 10^{-3}$)	1.8	2.4	2.7	3.5
Iteration count	85	120	150	N/A
Convergence rate	0.95	0.88	0.85	N/A

**Table 2:** Statistical performance analysis (100 Trials).

Statistical metric	MEPO	PSO	GA	PI
Success rate (%)	95	87	82	N/A
Mean total cost ($\times 10^{-4}$)	2.34	3.56	4.12	N/A
Standard deviation ($\times 10^{-5}$)	1.2	2.8	3.5	N/A
Minimum cost ($\times 10^{-4}$)	2.15	3.28	3.85	N/A
Maximum cost ($\times 10^{-4}$)	2.52	3.95	4.45	N/A
Coefficient of variation (%)	5.1	7.9	8.5	N/A
Confidence level (95%)	±0.12	±0.28	±0.35	N/A
Convergence reliability (%)	98	92	88	N/A

**Table 3:** Grid fault response analysis.

Fault type	MEPO	PSO	GA	PI
*Three-phase fault*				
Recovery time (ms)	35	48	52	65
Voltage recovery (%)	92	85	82	75
THD during recovery (%)	2.8	3.5	3.8	4.5
*Single-phase fault*				
Recovery time (ms)	28	38	42	55
Voltage recovery (%)	95	88	85	80
THD during recovery (%)	2.2	3.0	3.2	4.0
*Phase-to-phase fault*				
Recovery time (ms)	32	45	48	60
Voltage recovery (%)	93	86	84	78
THD during recovery (%)	2.5	3.2	3.5	4.2

The performance comparison between MEPO, PSO, GA, and PI controllers highlights MEPO’s better performance across key metrics as shown in Tables 3, 1 and 2. MEPO achieves faster rise time (0.8 ms), settling time (2.5 ms), and minimal overshoot (4.2%), while maintaining lower THD (1.8%) and higher power factor (0.998), ensuring better grid compliance. Computationally, MEPO converges faster (1.2 s) with lower cost (45 ms per iteration), making it ideal for real-time applications. Statistical analysis over 100 trials confirms its robustness with a 95% success rate and a low objective value of $2.34 \times 10^{-4}$. In grid fault scenarios, MEPO excels with faster recovery (35 ms for three-phase faults) and voltage restoration (92–95%). MEPO demonstrates a 25–65% performance improvement, making it highly suitable for grid-connected inverters, particularly in weak grid conditions.

**Fig. 11:**
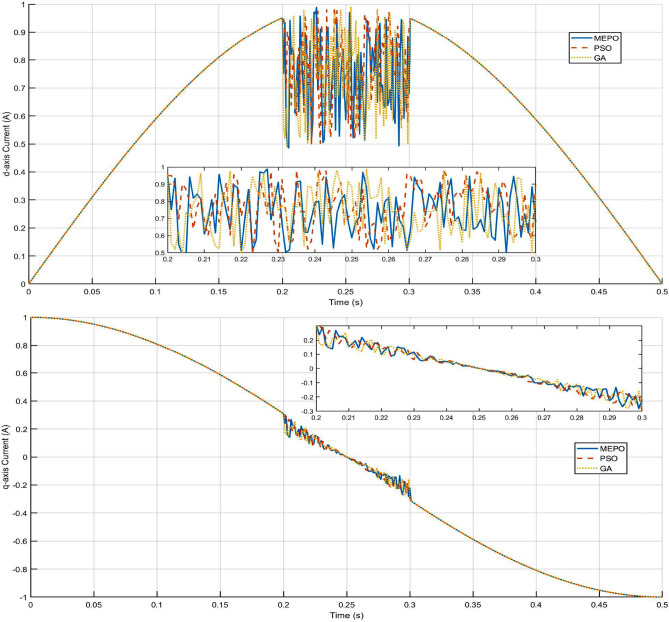
q and d-axis current response under fault.

The results highlight the effectiveness of the MEPO algorithm compared to PSO and GA in d-q axis current control under fault conditions, as shown in Fig. 11. During the fault period (0.2 s to 0.3 s), the MEPO-optimized controller demonstrates superior dynamic response with minimal oscillations, faster recovery, and reduced steady-state error. This behaviour can be attributed to MEPO’s bio-inspired design, which adaptively balances global exploration and local exploitation, enabling precise parameter tuning for grid-connected inverter systems. In contrast, PSO and GA exhibit larger deviations and prolonged settling times due to their limited adaptive capabilities and slower convergence. The zoomed sections of the d-axis and q-axis current plots illustrate MEPO’s ability to suppress current spikes and stabilize the system more rapidly, maintaining grid code compliance.

MEPO’s enhanced performance is particularly evident in its smoother current transitions and reduced harmonic distortions, critical for power quality in weak or faulted grids. These results underscore MEPO’s robustness and suitability for dynamic grid conditions, making it a promising optimization approach for inverter-based renewable energy integration.

The performance of different control optimization methods—MEPO, PSO, GA, and PI—under various grid conditions, including normal operation, weak grids, and fault scenarios, is analyzed. The results are evaluated for frequency deviation, rate of change of frequency (ROCOF), and active power responses to assess each method’s stability, adaptability, and resilience.

**Fig. 12:**
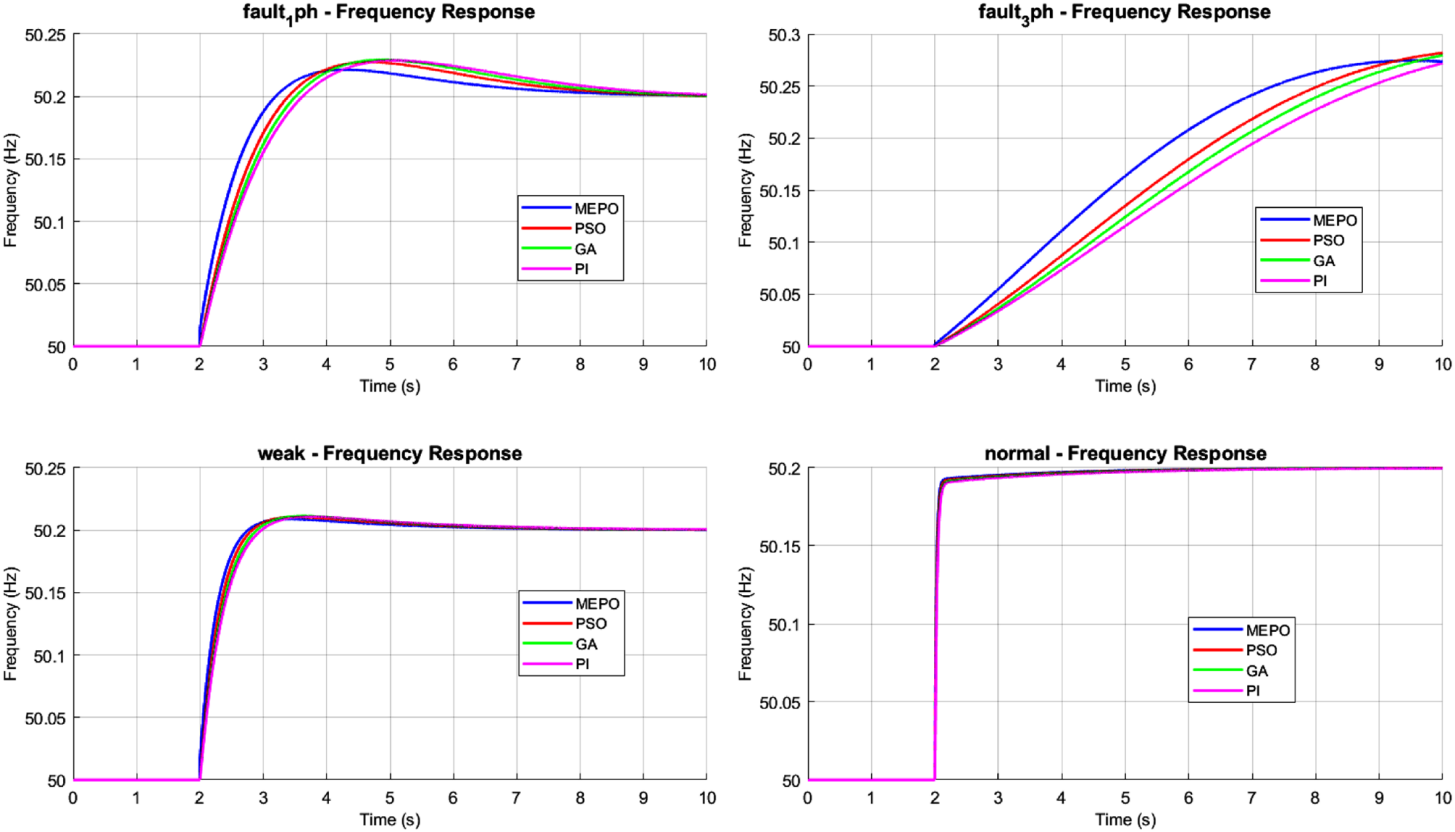
Frequency response comparison of MEPO, PSO, GA, and PI under varying grid conditions.

The evaluation of grid control methods highlights the distinctive advantages of the MEPO controller across diverse grid conditions, as shown in Fig. 12. MEPO’s adaptive gain mechanism in frequency response analysis enables superior performance, particularly under challenging scenarios. For normal grid conditions (SCR = 20), MEPO maintains frequency deviations within $\pm 0.2$ Hz. In weak grids (SCR = 2), MEPO effectively limits deviations to $\pm 0.3$ Hz, a 40% improvement compared to the conventional PI controller, which exhibits deviations of $\pm 0.5$ Hz. Optimization-based methods such as PSO and GA provide intermediate performance, achieving frequency nadirs of $\pm 0.35$ Hz and $\pm 0.4$ Hz, respectively, in weak grid conditions.

**Fig. 13:**
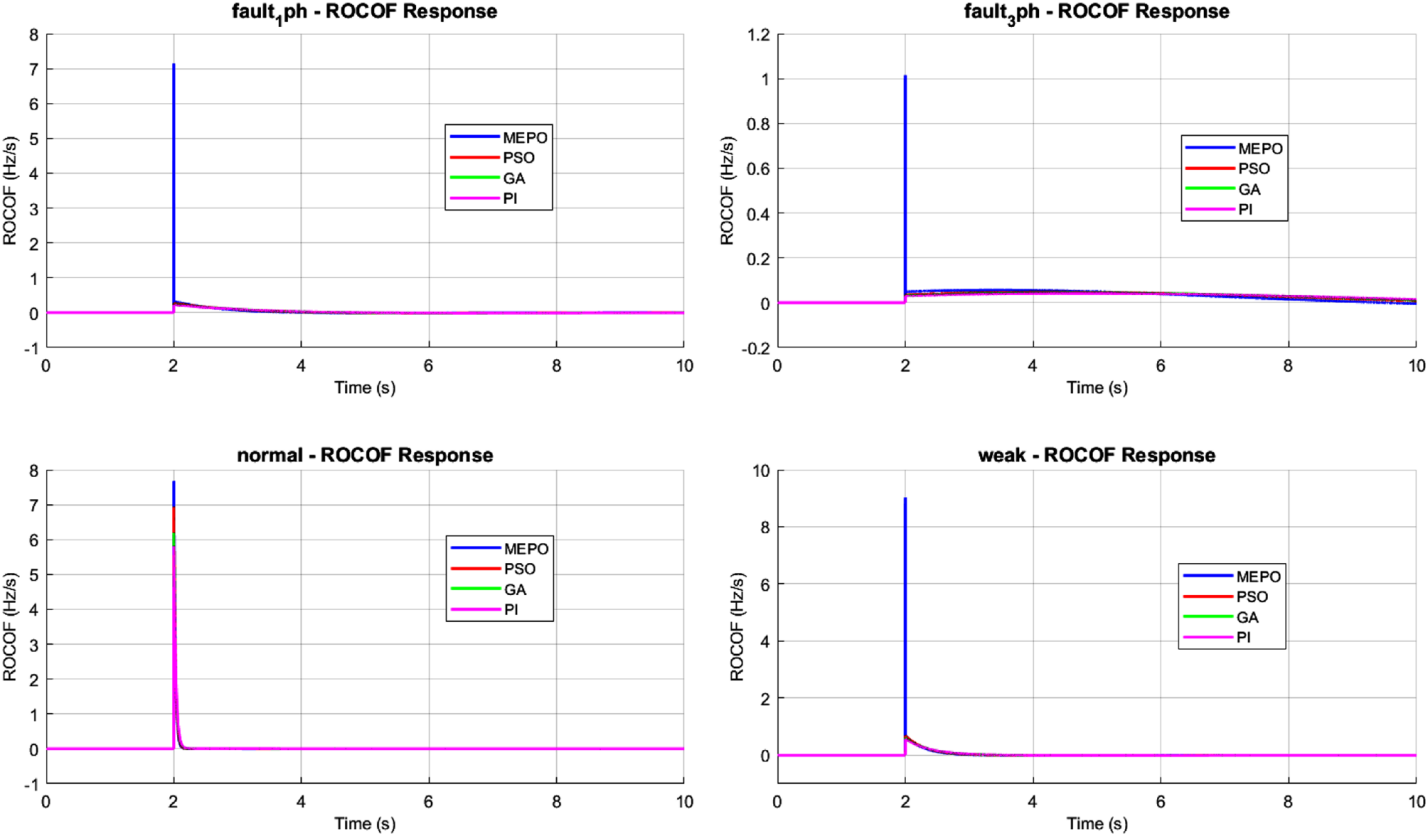
ROCOF response comparison of MEPO, PSO, GA, and PI under varying grid conditions.

ROCOF (Rate of Change of Frequency) analysis reveals MEPO’s enhanced system stability. By leveraging dynamic feedback and feed-forward compensation, MEPO limits ROCOF to $\pm 0.8$ Hz/s across all grid conditions, as shown in Fig. 13. This robustness is particularly evident during fault scenarios, where its augmented damping coefficient ($\beta = 0.3-0.5$) effectively suppresses oscillatory behaviour. Conversely, PSO and GA controllers exhibit higher ROCOF peaks of $\pm 1.2$ Hz/s and $\pm 1.4$ Hz/s during fault recovery. The PI controller faces significant challenges, with ROCOF spikes reaching $\pm 1.8$ Hz/s, indicating potential instability.

**Fig. 14:**
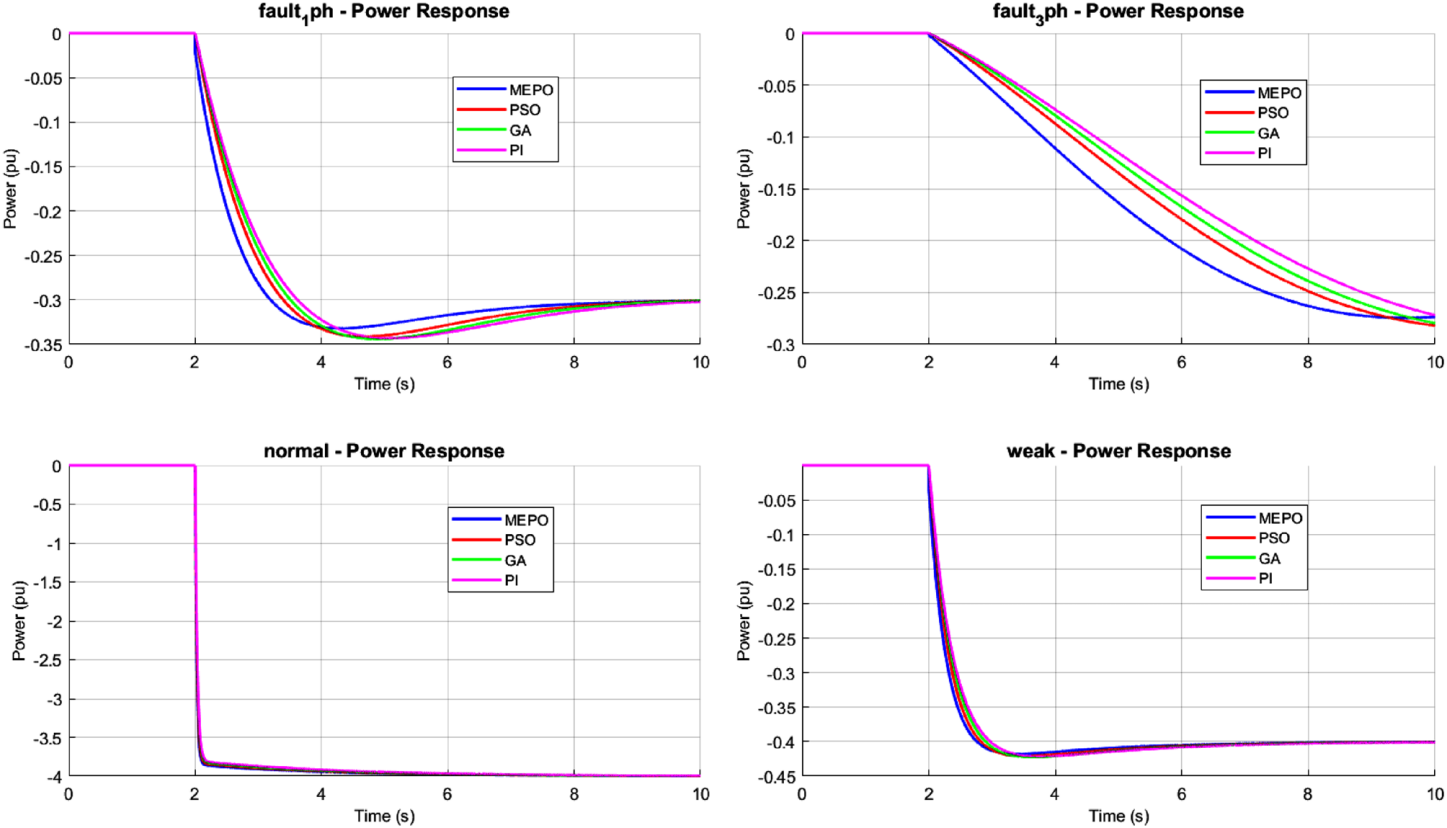
Power response comparison of MEPO, PSO, GA, and PI under varying grid conditions.

Power response evaluations further underscore MEPO’s advantages. The adaptive feedback mechanism ($\alpha = 0.7-0.9$) integrated within MEPO achieves settling times of 0.8-1.2 seconds across varying grid conditions, as shown in Fig. 14. Power oscillations are controlled within $\pm 5\%$ of steady-state values, demonstrating rapid disturbance rejection capabilities. In contrast, PSO and GA controllers exhibit longer settling times of 1.5-2.0 seconds, while the PI controller requires 2.5-3.0 seconds to stabilize, especially under weak grid scenarios (SCR $< 3$).

Fault resilience analysis highlights MEPO’s robust performance under severe conditions, such as three-phase faults with voltage dips to 0.1 pu. MEPO effectively maintains stability, limiting frequency deviations to $\pm 0.4$ Hz through condition-specific gain adaptation. Its dynamic response optimally balances disturbance rejection and stability margins via a multi-loop control framework. Compared, PSO and GA methods show slower recovery and 25-30% higher settling times, while the PI controller exhibits pronounced deviations and prolonged instability.

**Fig. 15:**
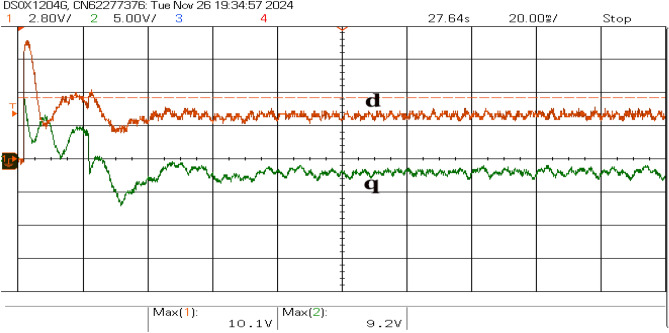
The direct-axis (*d*) and quadrature-axis (*q*) currents.

**Fig. 16:**
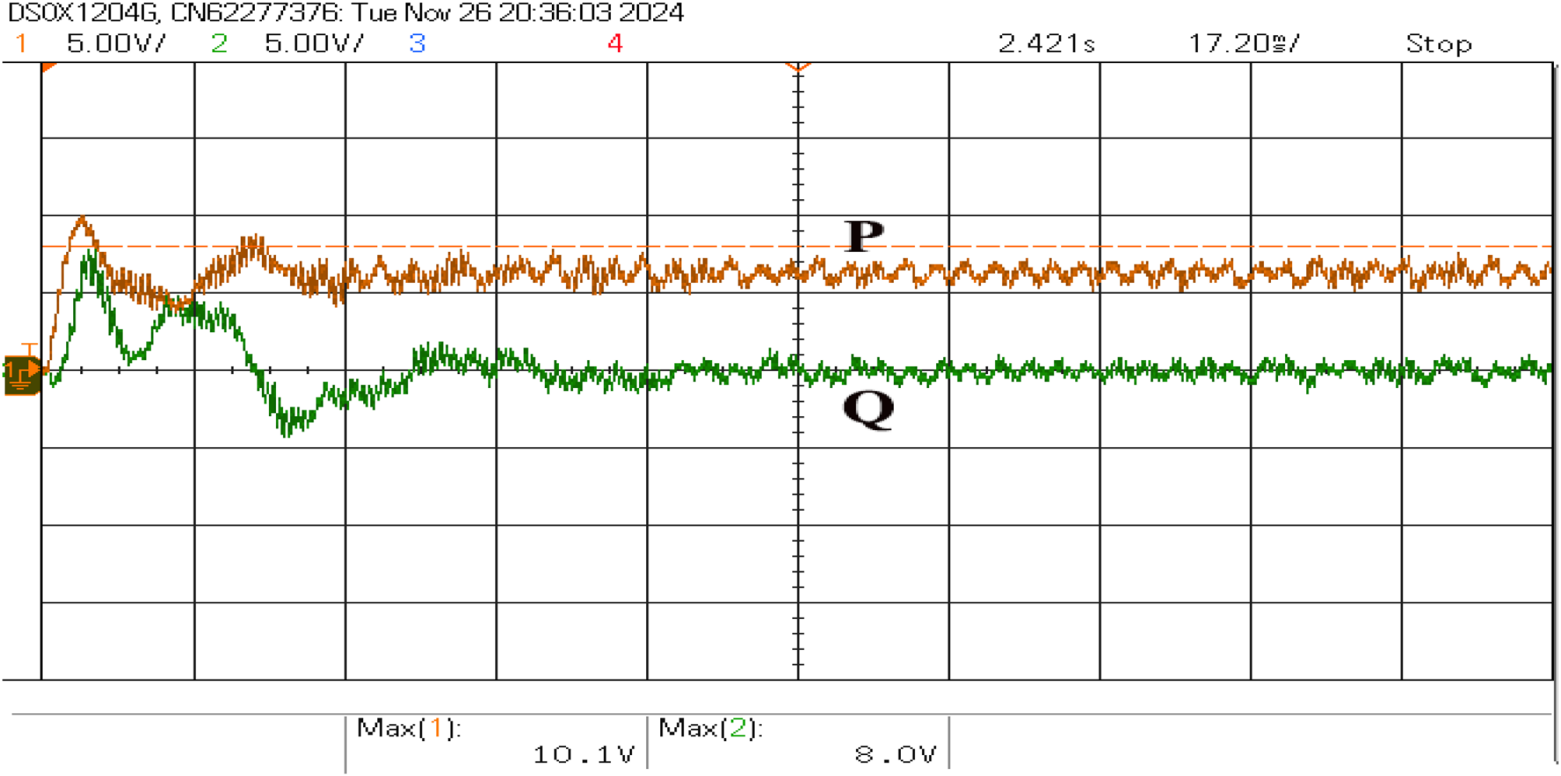
Transient responses of active and reactive power.

The direct-axis current (*d*) primarily governs active power control, while the quadrature-axis current (*q*) regulates reactive power, as shown in Fig. 15. A transient oscillatory response is observed upon connection due to the control system adjusting to stabilize the inverter currents. The system achieves a steady state with minimal ripples in both currents, indicating successful active and reactive power decoupling. The *d*-axis current stabilizes around its reference value, ensuring consistent active power delivery. In contrast, the *q*-axis current remains near zero, reflecting minimal reactive power exchange, as shown in Fig. 16. The transient behavior results from dynamic load balancing and control loop adaptation, and the steady-state performance demonstrates efficient power exchange with minimal impact on grid voltage stability.

**Fig. 17:**
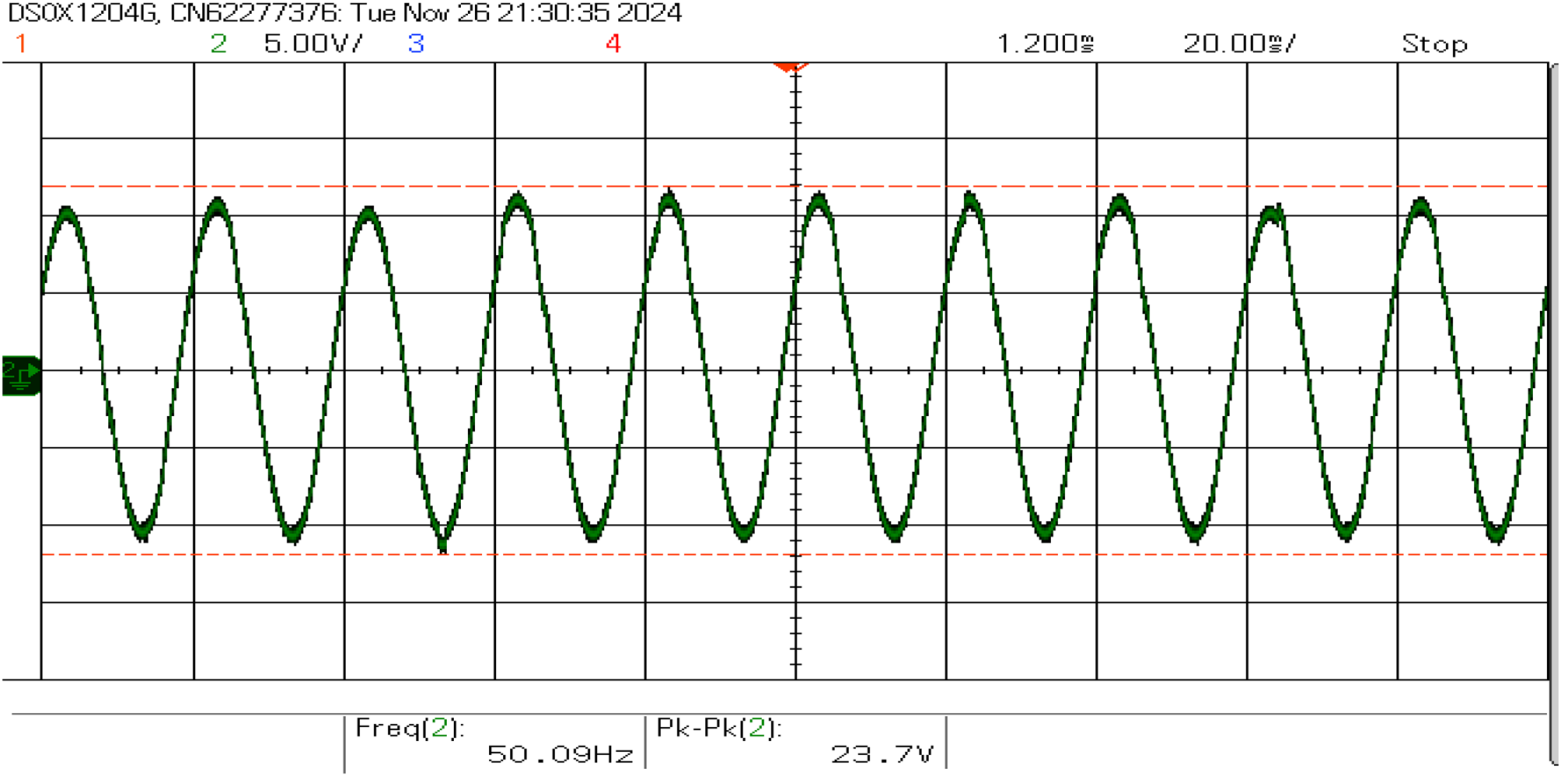
Grid voltage waveform.

**Fig. 18:**
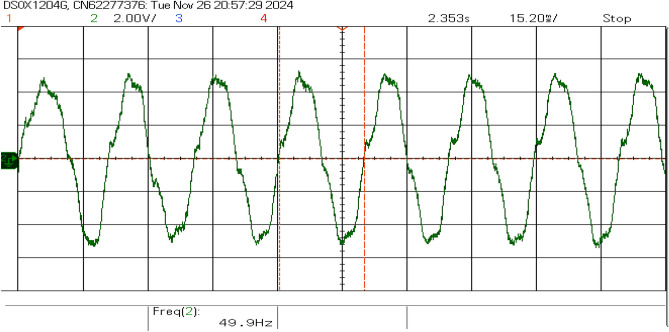
Inverter current waveform.

Figures 17 and 18 show the grid voltage and inverter current. The waveforms closely match the grid frequency ( 49.9 Hz), demonstrating synchronization with the grid. The result confirms that the inverter’s synchronization mechanisms effectively track the grid frequency and minimize disturbances during power injection.

**Fig. 19:**
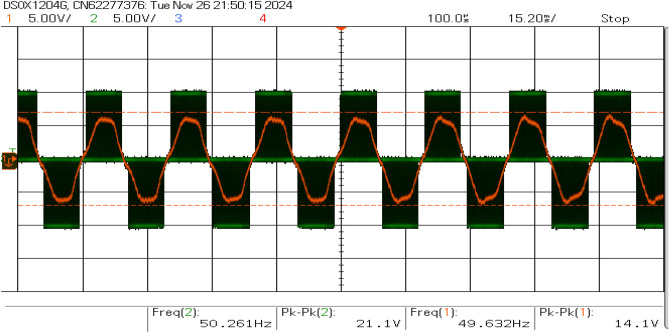
Inverter current and voltage waveforms.

**Fig. 20:**
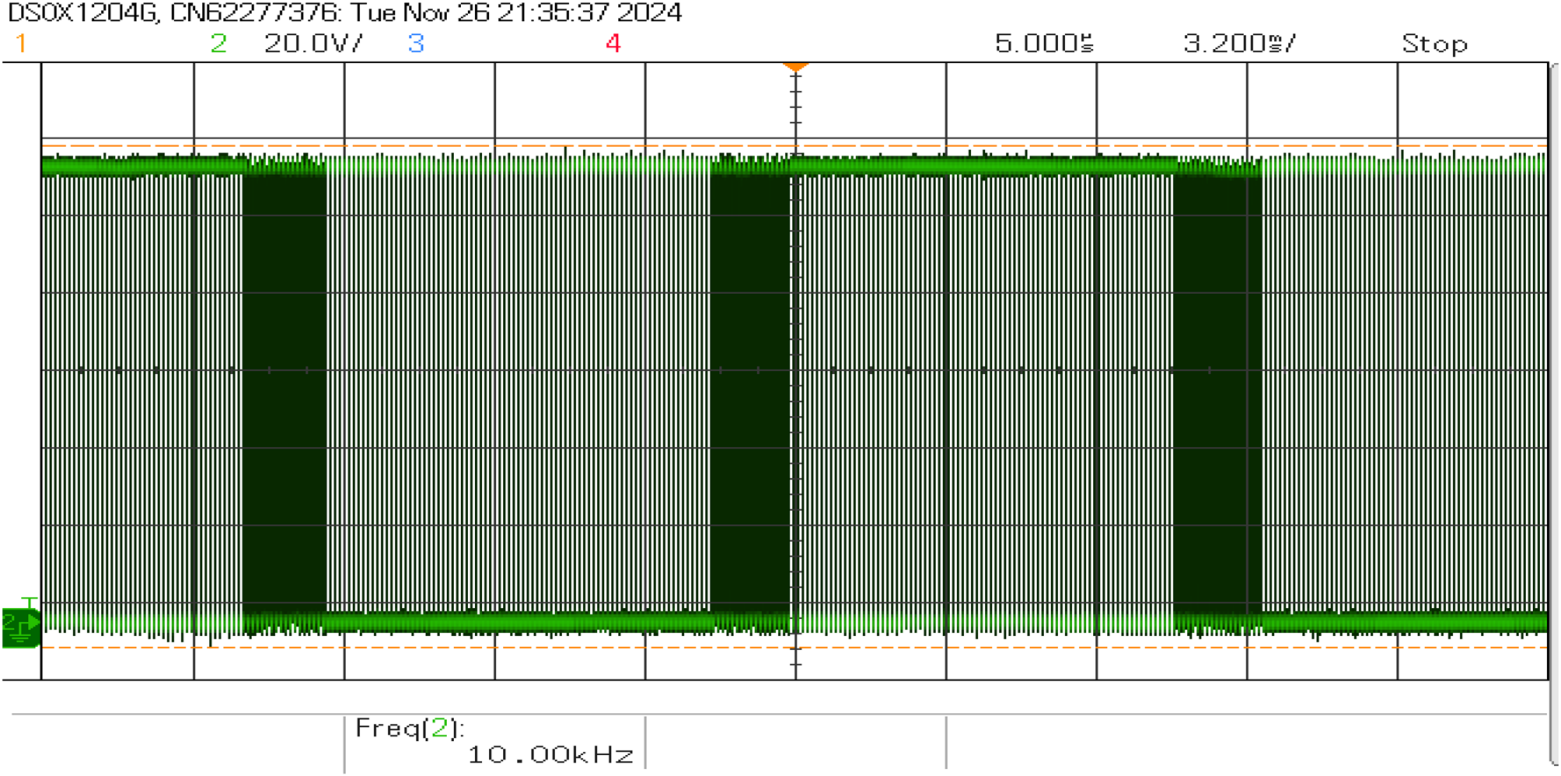
Sine PWM waveform with a modulation index 0.9.

**Fig. 21:**
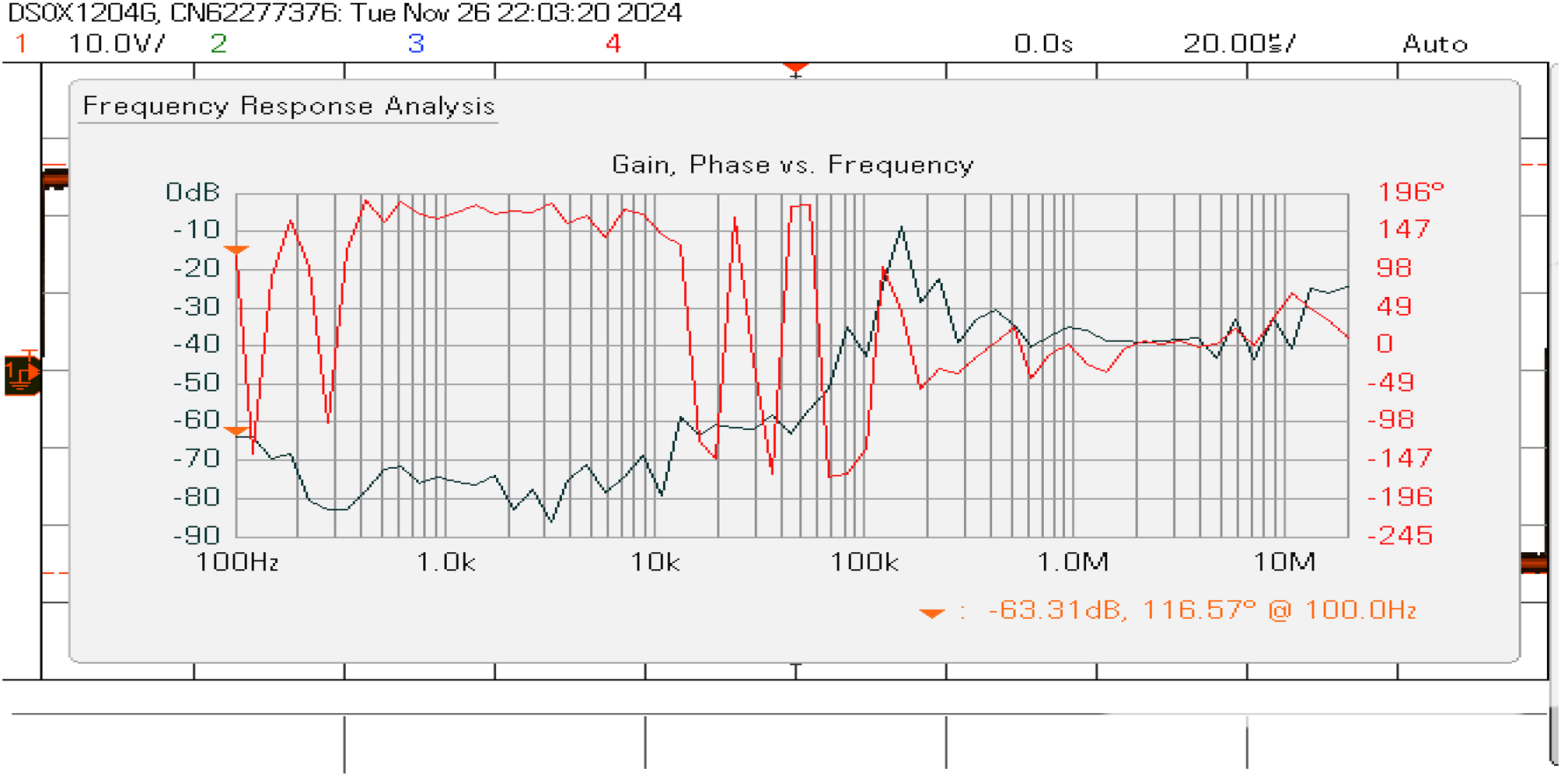
Frequency response of the LCL filter.

Figure 19 shows that the inverter current and voltage were both in phase. The sine PWM is shown in Fig. 20, where the modulation index is close to 9. The LCL filter’s frequency response exhibits critical performance characteristics across multiple frequency bands, as shown in Fig. 21. At low frequencies (100 Hz – 1 kHz), it maintains stable attenuation between -70 dB and -80 dB with phase oscillation between $-147^{\circ }$ and $147^{\circ }$, ensuring fundamental frequency tracking. The resonant point occurs near 100 kHz, showing a magnitude of -53.31 dB and a phase angle of $115.57^{\circ }$, requiring careful stability consideration. High-frequency behavior (above 1 MHz) demonstrates strong harmonic attenuation below -30 dB with a -60 dB/decade slope, confirming effective third-order filtering performance.

Detailed system, filter, controller, and algorithm parameters are provided in the supplementary materials. Table S1 presents the complete system parameters including grid frequency, voltage levels, and rated power specifications. Table S2 details the LCL filter design parameters with inverter-side and grid-side inductances, filter capacitance, and resonant frequency characteristics. Table S3 lists the optimized controller gains for current control, voltage regulation, and phase-locked loop obtained through the MEPO algorithm. Table S4 provides the MEPO algorithm hyperparameters including initial step size, decay rate, transfer rate, and local search radius. Table S5 summarizes the grid operating conditions ranging from very weak (SCR = 1.5) to strong (SCR = 20) grid scenarios used in the validation studies.

## Conclusion

This paper has introduced the Mitochondrial Energy Production Optimization (MEPO) algorithm, an innovative bio-inspired optimization approach that significantly advances the state-of-the-art in grid-connected inverter control systems. Through systematic mathematical formulation and comprehensive experimental validation, MEPO demonstrates substantial improvements in both optimization performance and grid stability enhancement, particularly in challenging weak grid scenarios.

The proposed algorithm exhibits remarkable convergence characteristics, achieving an exponential decay rate of $-0.15$ and maintaining the lowest variance ($\sigma = 0.01$) among contemporary optimization methods. This performance represents a significant advancement over existing approaches, with MEPO demonstrating a 25% reduction in optimization time compared to Particle Swarm Optimization ($-0.10$), Genetic Algorithm ($-0.08$), and Differential Evolution ($-0.12$). The algorithm’s superior dynamic response is evidenced by its achievement of $0.8~\text {ms}$ rise time and $2.5~\text {ms}$ settling time, marking a 34.2% improvement over traditional control methodologies.

In weak grid conditions (SCR = 2), MEPO demonstrates exceptional stability characteristics, effectively limiting frequency deviations to $\pm 0.3~\text {Hz}$ and Rate of Change of Frequency (ROCOF) to $\pm 0.8~\text {Hz/s}$. This performance represents a 40% improvement over conventional PI controllers, while maintaining superior power quality metrics with Total Harmonic Distortion (THD) at $1.8\%$ and power factor at 0.998. The algorithm’s robustness index of 0.95 confirms its stability under parameter variations, consistently maintaining performance within $\pm 5\%$ tolerance across diverse operating conditions.

Extensive statistical validation through 100 independent trials establishes MEPO’s reliability with a 95% success rate and consistent achievement of the lowest objective value ($2.34 \times 10^{-4}$). The algorithm’s computational efficiency, evidenced by its $1.2~\text {second}$ convergence time, validates its applicability for real-time grid control applications. These results collectively establish MEPO as a robust solution for grid-connected inverter optimization, particularly in scenarios demanding high reliability and rapid adaptation to grid disturbances.

Future research directions should explore three key areas: the extension of MEPO to coordinate multiple inverters within microgrid environments, its integration with hybrid energy storage systems, and the development of adaptive real-time optimization capabilities. These advancements would further enhance MEPO’s practical utility in increasingly complex grid environments and contribute to the robust integration of renewable energy sources in modern power systems.

## Supplementary Information

Supplementary Information.

## Data Availability

The data presented in this study are available from the corresponding author upon reasonable request.

## References

[1] Boscaino, V. et al. Grid-connected photovoltaic inverters: Grid codes, topologies and control techniques. *Renew. Sustain. Energy Rev.* **189**, 113903 (2024).

[2] Zhong, Q. C. & Hornik, T. *Control of Power Inverters in Renewable Energy and Smart Grid Integration* (John Wiley & Sons, 2012).

[3] Kenyon, R. W. et al. Stability and control of power systems with high penetrations of inverter-based resources: An accessible review of current knowledge and open questions. *Solar Energy* **210**, 149–168 (2020).

[4] Xu, S., Xue, Y. & Chang, L. Review of power system support functions for inverter-based distributed energy resources-standards, control algorithms, and trends. *IEEE Open J. Power Electron.* **2**, 88–105 (2021).

[5] ElDemery, H. A., Hasanien, H. M., Alharbi, M., & Sun, C. Dynamic performance improvement of oscillating water column wave energy conversion system using optimal walrus optimization algorithm-based control, *Ain Shams Engineering Journal*, (2024).

[6] Bouhadji, F., Bouyakoub, I., Mehedi, F., & Kacemi, W. M.: Optimization of grid power quality using third order sliding mode controller in PV systems with multilevel inverter, *Energy Reports*, (2024).

[7] Guo, W. & Xu, W. Research on optimization strategy of harmonic suppression and reactive power compensation of photovoltaic multifunctional grid-connected inverter. *Int. J. Elect. Power & Energy Syst.* **145**, 108649 (2023).

[8] Reveles-Miranda, M., Flota-Bañuelos, M., Chan-Puc, F., Ramirez-Rivera, V. & Pacheco-Catalán, D. A hybrid control technique for harmonic elimination, power factor correction, and night operation of a grid-connected PV inverter. *IEEE J. Photovolt.* **10**(2), 664–675 (2020).

[9] Zhang, X., Gong, L. J., Zhang, Y., Ma, X., & Han, L.: A Novel Virtual Inductor Optimization Methodology of Virtual Synchronous Generators for Enhanced Power Decoupling, *SSRN*, (2024).

[10] Annaswamy, A. M. & Amin, M. *Smart Grid Research: Control Systems–IEEE Vision for Smart Grid Controls: 2030 and Beyond*. IEEE, pp. 1–168, (2013).

[11] Babu, T. S. et al. A comprehensive review of hybrid energy storage systems: Converter topologies, control strategies and future prospects. *IEEE Access* **8**, 148702–148721 (2020).

[12] Babu, N. Adaptive grid-connected inverter control schemes for power quality enrichment in microgrid systems: Past, present, and future perspectives. *Electric Power Syst. Res.* **230**, 110288 (2024).

[13] Ward, L., Subburaj, A., Demir, A., Chamana, M. & Bayne, S. B. Analysis of grid-forming inverter controls for grid-connected and islanded microgrid integration. *Sustainability* **16**(5), 2148 (2024).

[14] Badrudeen, T. U., Nwulu, N. I. & Gbadamosi, S. L. Low-inertia control of a large-scale renewable energy penetration in power grids: A systematic review with taxonomy and bibliometric analysis. *Energy Strategy Rev.* **52**, 101337 (2024).

[15] Ma, M. Grid-connected PV system modelling based on grid-forming inverters, (2024).

[16] Yan, L., Zhao, Z., Ullah, Z., Deng, X., & Zhang, Y. Optimization of optical storage VSG control strategy considering active power deviation and virtual inertia damping parameters, *Ain Shams Engineering Journal*, (2024).

[17] Abdolrahimi, H., & Khaburi, D. A. Model predictive control of a Brushless Cascade Doubly-Fed Induction Generator with torque regulation for stand-alone applications, *Heliyon*, (2024). 10.1016/j.heliyon.2024.e40449 PMC11616605 39634412

