# Supplementary material for: Bio-inspired mitochondrial energy optimization for enhanced grid-connected inverter performance in weak grid systems
Source: Sci Rep. 2025 Dec 29;15:44836. doi: 10.1038/s41598-025-28884-9 (PMC12749809; doi:10.1038/s41598-025-28884-9)
Supplement: Supplementary file 1 — Supplementary Information. [file 41598_2025_28884_MOESM1_ESM.pdf]

Supplementary Materials

Bio-Inspired Mitochondrial Energy Optimization for Enhanced Grid-Connected Inverter Performance in Weak Grid Systems

Mrinal Kanti Rajak*
Rajen Pudur

Department of Electrical Engineering
National Institute of Technology Arunachal Pradesh
Jote, Arunachal Pradesh 791113, India

System Parameters

Table S1: System parameters used in the grid-connected inverter simulation and experimental setup.

**Parameter**	**Symbol**	**Value**	**Unit**	**Description**
Nominal grid frequency	f_{grid}	50	Hz	Fundamental grid frequency
Grid voltage (RMS)	V_{grid}	230	V	Nominal grid voltage
DC link voltage	V_{dc}	800	V	Inverter DC bus voltage
Switching frequency	f_{sw}	10	kHz	Inverter switching frequency
Sampling time	T_{s}	1×10^{-5}	s	Control sampling period
Rated power	S_{rated}	10	kW	System rated power

*Corresponding author. Email: mrinal.phd20@nitap.ac.in

Filter Parameters

Table S2: LCL filter design parameters for harmonic attenuation and grid interface.

Parameter	Symbol	Value	Unit	Description
Inverter-side inductance	L_1	2	mH	LCL filter inverter inductance
Grid-side inductance	L_2	1	mH	LCL filter grid inductance
Filter capacitance	C	10	μ F	LCL filter capacitance
Filter resistance	R	0.1	Ω	Damping resistance
Resonant frequency	f_{res}	100	kHz	LCL filter resonance

Controller Parameters

Table S3: Optimized controller gains for current control, voltage regulation, and phase-locked loop obtained through MEPO algorithm.

Parameter	Symbol	Value	Unit	Description
Current proportional gain	K_{p_i}	45.3	-	Current controller P gain
Current integral gain	K_{i_i}	892.7	-	Current controller I gain
Voltage proportional gain	K_{p_v}	12.8	-	Voltage controller P gain
Voltage integral gain	K_{i_v}	156.4	-	Voltage controller I gain
PLL proportional gain	$K_{p_{\text{pll}}}$	4.2	-	PLL P gain
PLL integral gain	$K_{i_{\text{pll}}}$	65.7	-	PLL I gain

MEPO Algorithm Parameters

Table S4: MEPO algorithm hyperparameters for optimization convergence and exploration-exploitation balance.

Parameter	Symbol	Value	Unit	Description
Initial step size	η_0	0.1	-	Starting learning rate

Continued on next page

Table S4 – continued from previous page

Parameter	Symbol	Value	Unit	Description
Decay rate	λ	0.01	-	Step size decay parameter
Transfer rate	r_{et}	0.3	-	Electron transfer probability
Local search radius	σ_0	0.01	-	Initial search variance

Grid Conditions

Table S5: Grid operating conditions ranging from very weak (SCR = 1.5) to strong (SCR = 20) grid scenarios.

Parameter	Symbol	Value	Unit	Description
Short circuit ratio	SCR	1.5–20	-	Grid strength indicator
X/R ratio	X/R	2–10	-	Grid impedance characteristic
Voltage variation	ΔV	±10%	-	Allowed voltage range
Frequency deviation	Δf	±0.5	Hz	Allowed frequency range
